# Supplementary material for: Estimating subnational excess mortality in times of pandemic. An application to French départements in 2020
Source: PLoS One. 2024 Jan 19;19(1):e0293752. doi: 10.1371/journal.pone.0293752 (PMC10798530; doi:10.1371/journal.pone.0293752)
Supplement: S1 Appendix — Presents derivations for computing, from CP-spline estimated coefficients and observed mortality rates, excess mortality measured by different demographic indicators. (PDF) [file pone.0293752.s001.pdf]

Estimating Subnational Excess Mortality in Times of Pandemic.
S1 Appendix

October 30, 2023

Methods for computing excess mortality

As stated in the paper, common concepts can be used for all demographic indicators when employed for measuring excess mortality. Firstly, the baseline mortality, which represents the mortality that would have been observed in the absence of a pandemic, is fully characterized by the coefficients vector $\boldsymbol{\alpha}$, estimated through CP-splines (equation (1) in the manuscript). Secondly, the level of mortality observed in 2020 is described by the series of observed rates $\boldsymbol{m}_{2020}$. Combining these two sources of uncertainty when measuring excess mortality is paramount when dealing with small spatial units.

Equations presented in Section 2 of the paper can be adapted for life expectancy at any age x by modifying vector $\mathbf{1}_m$ and matrix $\boldsymbol{C}$, i.e. replace with zeros rows/columns corresponding to ages smaller than x. Similar arguments can be applied to age-standardized mortality rates and plain death toll presented below.

Lastly, we provide a clear and explicit definition of the matrix $\boldsymbol{L}$ utilized in the paper and in subsequent sections of this document. Given a mn-vector of mortality $\boldsymbol{\mu}$ for m ages and n years, the $m \times mn$ matrix $\boldsymbol{L}$ is employed to selectively obtain the forecasted mortality for the year 2020 and all ages.

$$\boldsymbol{L} = \left[\ \mathbf{0}_{m\times m(n-1)} : \ \ \boldsymbol{I}_m \ \right] , \tag{1}$$

where $\boldsymbol{I}_m$ is the identity matrix of dimension m and the other element is a matrix of zeros with the associated dimensions.

The versatility of this matrix allows for its adaptation to select other specific years by incorporating the identity matrix in particular parts of $\boldsymbol{L}$. The significance of this matrix lies in the fact that the entire forecasting process encompasses all available ages and years. As a result, we require a formulation of the associated measures (e.g., life expectancy) that incorporates estimated mortality across all ages and years, and $\boldsymbol{L}$ allows us to obtain it.

Age-standardized death rate

An age-standardized mortality rate (SDR) is the crude death rates of a population of size κ adjusted to a standard age distribution, $\boldsymbol{p}$, and it is calculated as a weighted average

of the age-specific mortality rates. Excess mortality measured by SDR, the analogous of equation (2) in the paper for this indicator, is given by

$$\delta_{\mathrm{SDR}} = \mathrm{SDR}^F(\boldsymbol{\alpha}) - \mathrm{SDR}^O(\boldsymbol{m}_{2020}) = \kappa * (\boldsymbol{p} * \mathbf{1}_m)'(\boldsymbol{L}\boldsymbol{\mu} - \boldsymbol{m}_{2020})\,, \tag{2}$$

where $*$ denote element-wise product. In the available routines, European Population Standard of 2013 (?) is provided for $\boldsymbol{p}$.

The variability associated with both the observed and forecasted SDR in 2020 can be determined using equation (3) from the paper by substituting the partial derivatives with the following equations

$$\begin{aligned} \nabla\mathrm{SDR}^F(\boldsymbol{\alpha}) &= \kappa * (\boldsymbol{B}'\,(\boldsymbol{L}'\,(\boldsymbol{p} * \mathbf{1}_m) * \boldsymbol{\mu}))' \\ \nabla\mathrm{SDR}^O(\boldsymbol{m}_{2020}) &= \kappa * (\boldsymbol{p} * \mathbf{1}_m * \boldsymbol{m}_{2020})'\,, \end{aligned} \tag{3}$$

and then combine the variances from both sources to calculate the overall uncertainty surrounding excess mortality as measured by SDR.

Death toll

Excess mortality can be calculated by subtracting the projected total number of deaths that would have occurred in the absence of a pandemic from the actual reported death toll (DT) in the pandemic year (in this case, 2020). While life expectancy and SDR are age-standardized metrics, the total number of deaths during a pandemic is influenced by the population's structure and size. Nevertheless, this measure is easily comprehensible and accessible, even for non-experts, and it offers a direct assessment of the actual burden of the pandemic in a specific region.

Computed as follows:

$$\delta_{\mathrm{DT}} = \mathrm{DT}^F(\boldsymbol{\alpha}) - \mathrm{DT}^O(\boldsymbol{m}_{2020}) = \mathbf{1}_m\,[\boldsymbol{L}(\boldsymbol{e} * \boldsymbol{\mu}) - \boldsymbol{d}_{2020}]\,, \tag{4}$$

the partial derivatives of this measure for both baseline and observed mortality are given by

$$\begin{aligned} \nabla\mathrm{DT}^F(\boldsymbol{\alpha}) &= (\boldsymbol{B}'\,(\boldsymbol{L}'\,\mathbf{1}_m * \boldsymbol{e} * \boldsymbol{\mu}))' \\ \nabla\mathrm{DT}^O(\boldsymbol{m}_{2020}) &= (\mathbf{1}_m * \boldsymbol{d}_{2020})'\,. \end{aligned} \tag{5}$$
