# Supplementary material for: Estimating subnational excess mortality in times of pandemic. An application to French départements in 2020
Source: PLoS One. 2024 Jan 19;19(1):e0293752. doi: 10.1371/journal.pone.0293752 (PMC10798530; doi:10.1371/journal.pone.0293752)
Supplement: S2 Appendix — Presents a comparison between CP-spline and the Lee-Carter model of excess mortality estimates. (PDF) [file pone.0293752.s002.pdf]

Estimating Subnational Excess Mortality in Times of Pandemic.
S2 Appendix

October 30, 2023

Comparison with the Lee-Carter model

Instead of considering pre-pandemic mortality levels as the baseline in absence of COVID-19, we opted to forecast mortality in 2020 extrapolating mortality temporal changes. Among the numerous forecasting mortality methods, we rely on CP-splines (Camarda, 2019) given the numerous advantages in terms of statistical robustness and flexibility. However, the recent development of this methodology has prompted the reviewers to ask for a comparison to an alternative approach.

A comprehensive comparison of various mortality forecasting methods goes beyond the purpose of this paper and appendix, as it has already been covered elsewhere[1]. Here, our focus is on the Lee-Carter model (Lee and Carter, 1992), which serves as a benchmark approach for forecasting mortality. In a nutshell, the Lee-Carter model utilizes principal components to describe the evolution of mortality over age and time. This simplifies the problem from a two-dimensional perspective to standard mortality age-pattern, a fixed-age effect and a univariate time index. The time index captures historical changes in mortality, and it is extrapolated using time-series analysis to forecast future mortality. For a more thorough understanding of this model and its relevance in mortality forecasting, please refer to the work of Basellini et al. (2023).

In order to maintain a consistent and appropriate stochastic framework for mortality data, we made the decision to estimate the Lee-Carter model using a Poisson setting, as described in Brouhns et al. (2002). As a result, any discrepancies between the two approaches will solely arise from a different functional form used to describe mortality developments.

Figure 1 illustrates the comparison of the two approaches in terms of losses in male life expectancy at age 60 in 2020 for each French *département*. The outcomes show a considerable similarity, particularly for larger geographical subunits. With only a few exceptions, the losses estimated by the Lee-Carter model fall within the 95% confidence intervals of those estimated by CP-splines. As a result, we have confidence that employing a different approach would not significantly alter our final conclusions.

[1]See the extensive comparison in the original paper about CP-splines, and associated online supplementary material.

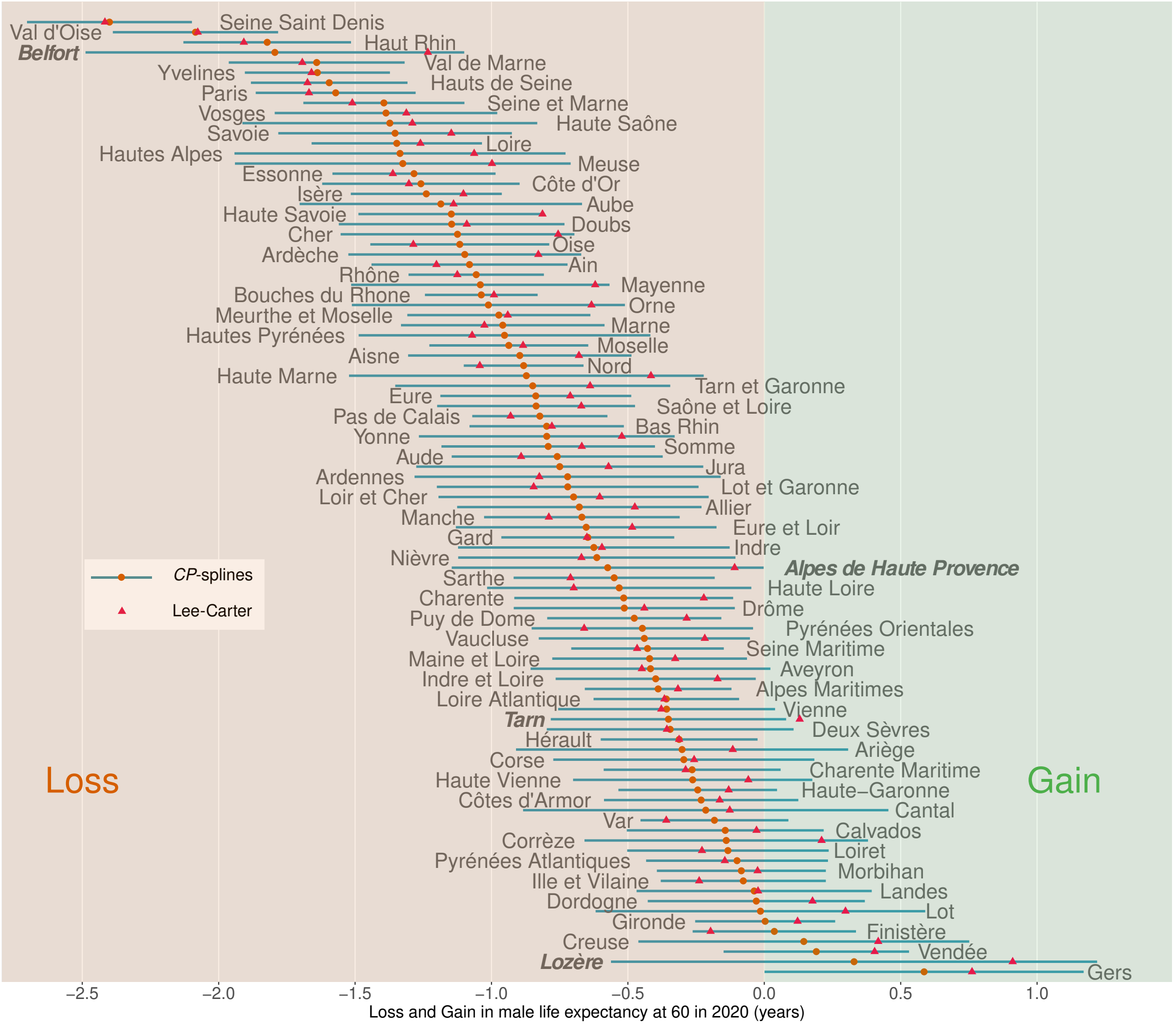

Figure 1: **Losses in male life expectancy at age 60 in 2020 for each French *département*.** Colored dots depict point-estimates for CP-splines (orange) and Lee-Carter model (red). Horizontal bars represent represent 95% level confidence interval for CP-splines. In bold the four *départements* with the largest differences between the two forecasting approaches.

To conduct a more detailed comparison, we focus on four *départements* where the discrepancies in losses between the Lee-Carter and CP-splines approaches are the largest: *Tarn*, *Lozère*, *Alpes de Haute Provence* and *Belfort*. These regions are depicted in bold in Figure 1. Figure 2 displays the observed and estimated life expectancy at age 60 for both methods in these specific geographical areas. As outlined in the paper, we optimize the starting period for each subunit, perform estimation up to 2019, and forecast for 2020. The 95% confidence intervals are depicted for both approaches.

While the CP-splines automatically provide uncertainty measures for both observed and forecast years, the default Lee-Carter model only offers uncertainty measures for future years. Upon analyzing the figure, it becomes evident that the Lee-Carter model is overfitting the e_{60} data, and its forecast can be regarded as somewhat inaccurate. Consequently, the relatively large differences in losses compared to the CP-spline estimates can

be attributed to the subpar performance of the Lee-Carter model in these *départements*.

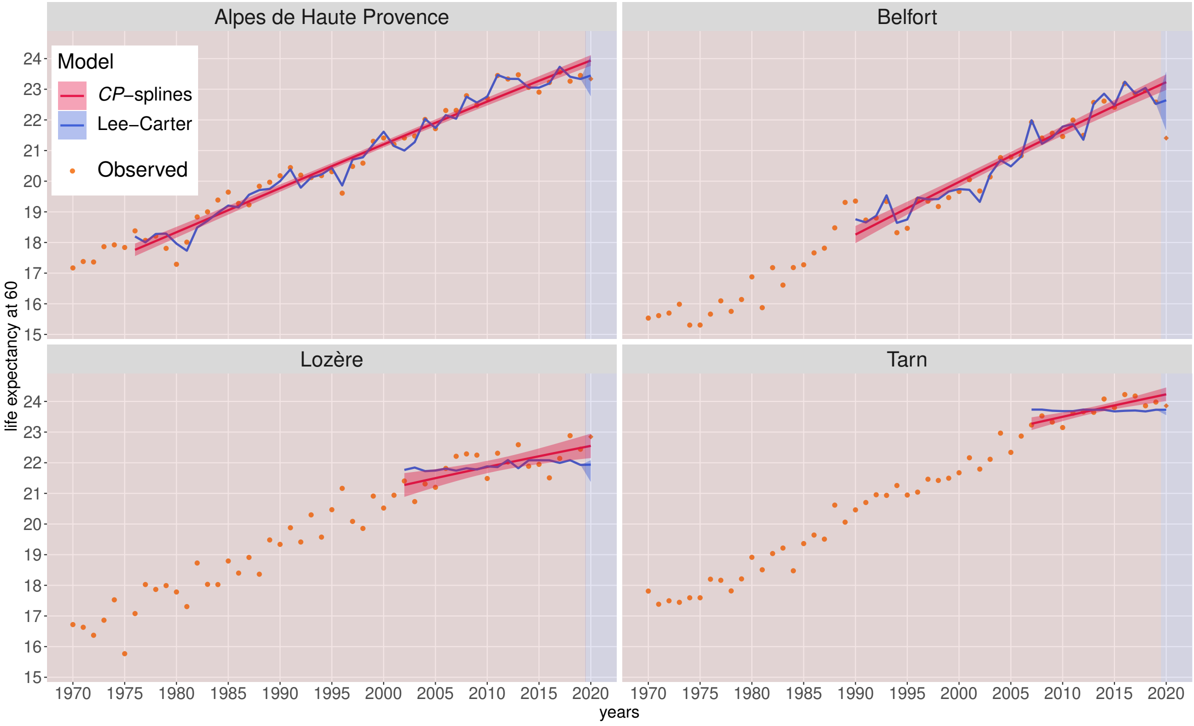

Figure 2: **Observed, estimated and forecast life expectancy at age 60, produced by both the CP-splines and Lee-Carter methods for four specific French *départements* where the differences in estimated 2020 losses in e_{60} between the two approaches are the largest.** Starting year optimized for each *département*, estimated up to 2019 and forecast in 2020.

The proposed model exhibits an additional advantage that becomes apparent when examining the mortality age-pattern for a specific year. In Figure 3, both the observed and forecast log-rates in 2020 are depicted for the mentioned four *départements*, using both the Lee-Carter and CP-splines approaches. The Lee-Carter estimates display a wiggling behavior, in stark contrast to the smoother patterns fitted by CP-splines.

Despite the Lee-Carter model's unreasonableness, summary measures like life expectancy might conceal these erratic forecast age-patterns. However, when losses are measured by indicators for specific ages, this erratic behavior can have a detrimental effect. In contrast, CP-splines demonstrate robustness and are exempt from such unrealistic behaviors.

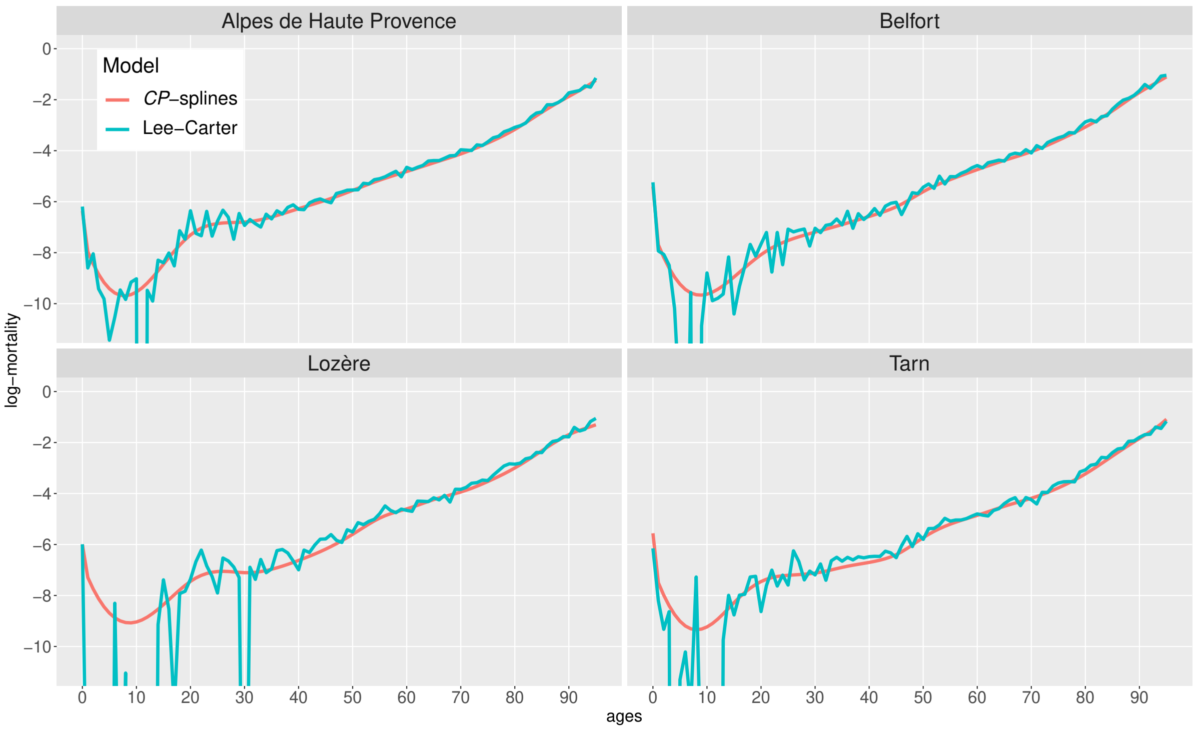

Figure 3: **Forecast age-specific log-rate for the year 2020, produced by both the CP-splines and Lee-Carter methods for four specific French *départements* where the differences in estimated 2020 losses in e_{60} between the two approaches are the largest.**

References

Basellini, U., Camarda, C. G., and Booth, H. (2023). Thirty years on: A review of the Lee-Carter method for forecasting mortality. *International Journal of Forecasting.*, 39:1033–1049.

Brouhns, N., Denuit, M., and Vermunt, J. K. (2002). A Poisson log-bilinear regression approach to the construction of projected lifetables. *Insurance: Mathematics & Economics*, 31:373–393.

Camarda, C. G. (2019). Smooth Constrained Mortality Forecasting. *Demographic Research*, 41(38):1091–1130.

Lee, R. D. and Carter, L. R. (1992). Modeling and Forecasting U.S. Mortality. *Journal of the American Statistical Association*, 87:659–671.
