# Supplementary material for: Estimating subnational excess mortality in times of pandemic. An application to French départements in 2020
Source: PLoS One. 2024 Jan 19;19(1):e0293752. doi: 10.1371/journal.pone.0293752 (PMC10798530; doi:10.1371/journal.pone.0293752)
Supplement: S3 Appendix — Presents additional figures, maps and tables of excess mortality estimates. (PDF) [file pone.0293752.s003.pdf]

Estimating Subnational Excess Mortality in Times of Pandemic.
S3 Appendix

January 9, 2024

Additional figures and tables

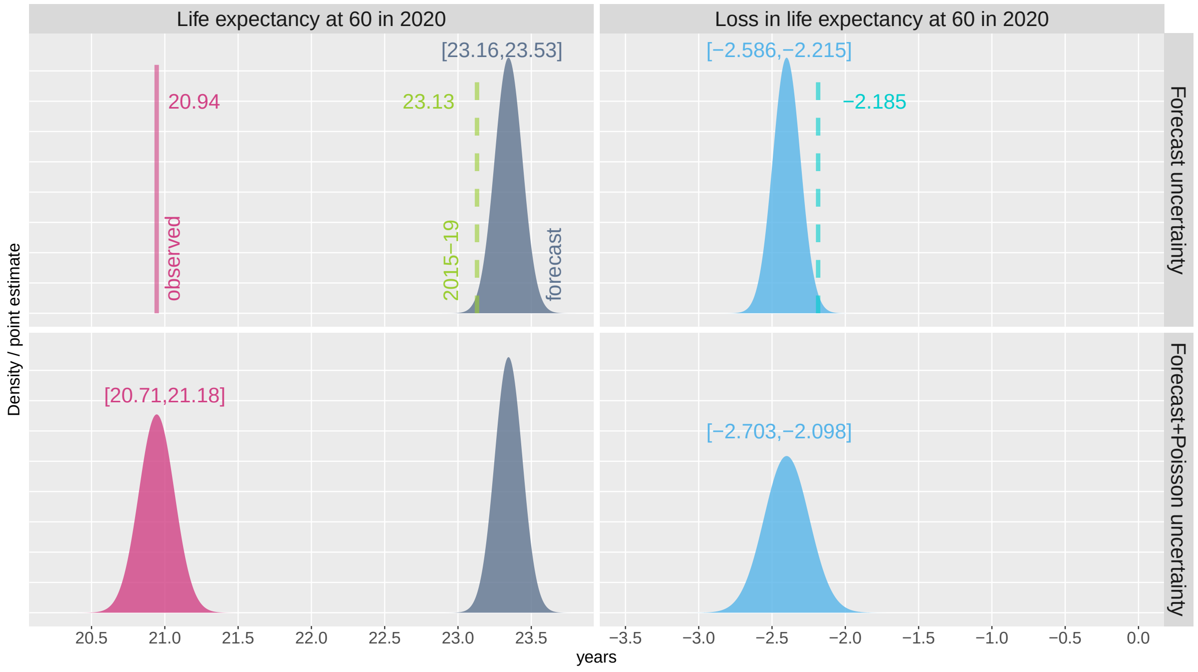

Figure 1: Illustrative figure of sources of uncertainty around excess mortality measure. Life expectancy at 60 (left panels) and associated losses (right panels) for *Seine-Saint-Denis*, males, 2020. Upper panels: forecast uncertainty is accounted. Lower panel: both forecast and Poisson uncertainty are reported. Texts refer to either point estimates or 95% confidence intervals. Dashed lines depict "simple" estimation of excess mortality.

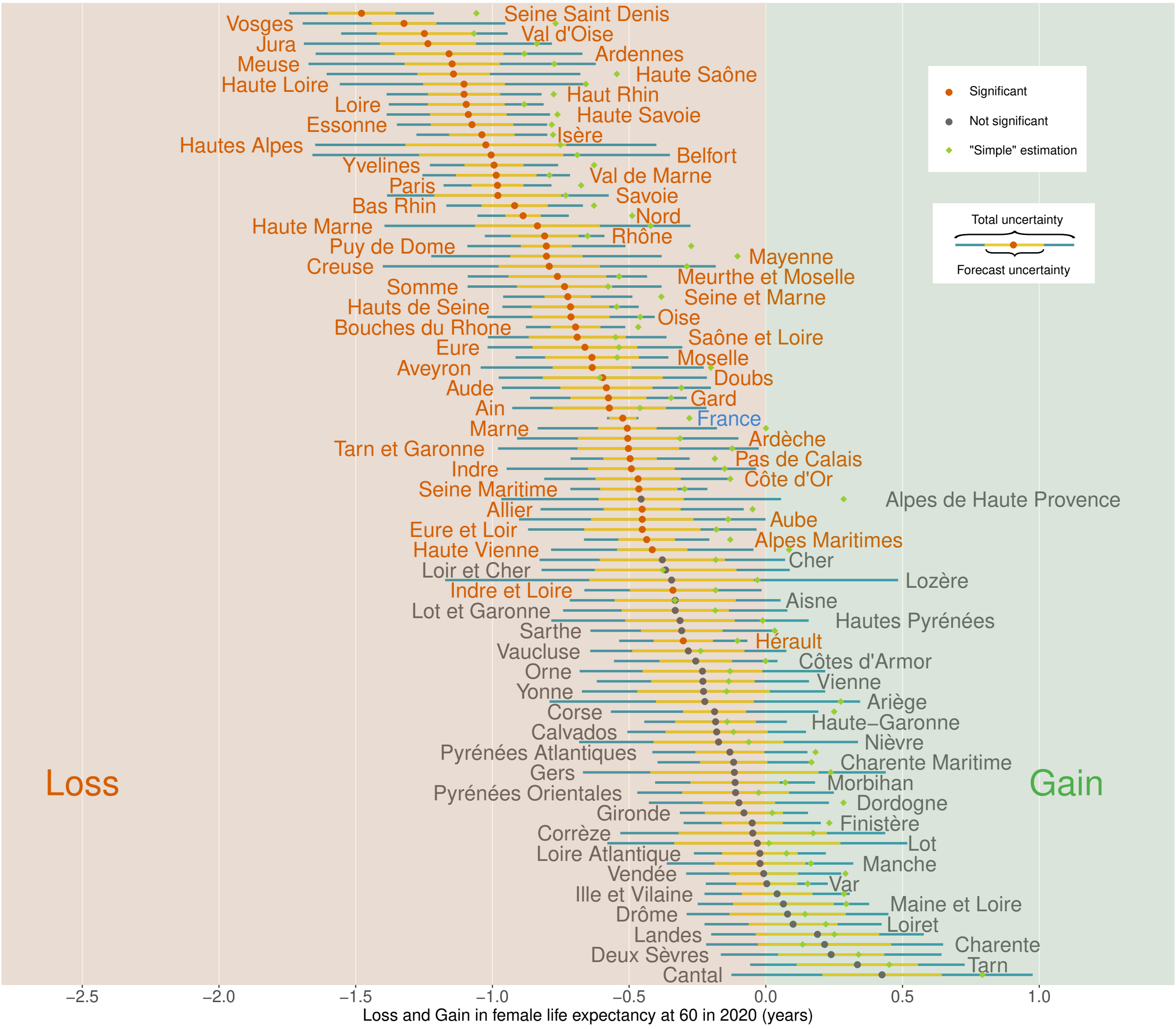

Figure 2: Losses in life expectancy at age 60 in 2020 for each French *département*, for females. Colors of dots and texts express the presence of significant estimates at 95% level, and colors of the horizontal bars represent the two sources of uncertainty. Green dots identify “simple” estimates of losses.

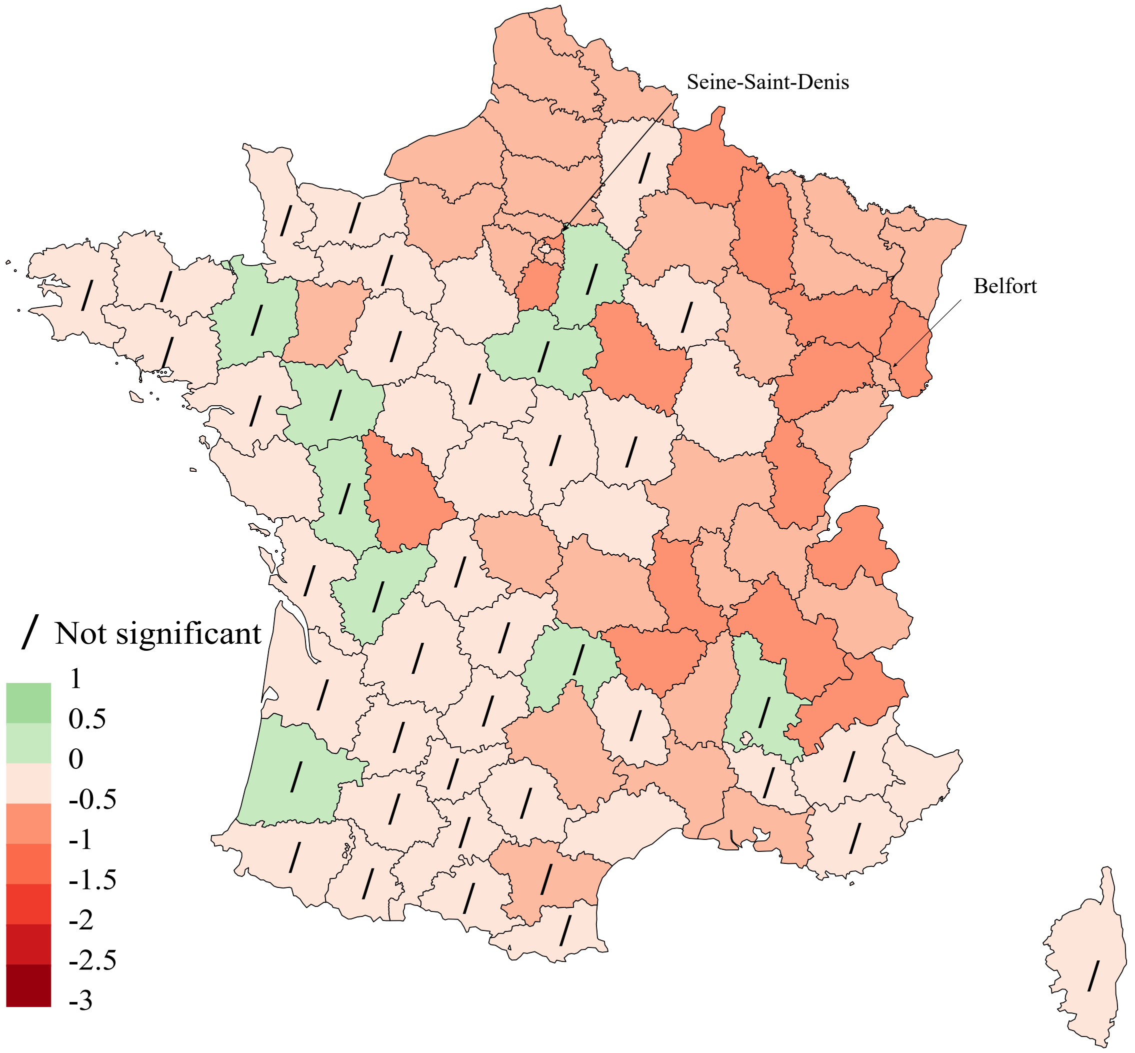

Figure 3: Map of France *départements* by losses/gains in female life expectancy at age 60 in 2020. Slash symbol (/) denote areas with loss/gain in e_{60} not significant at 5% level.

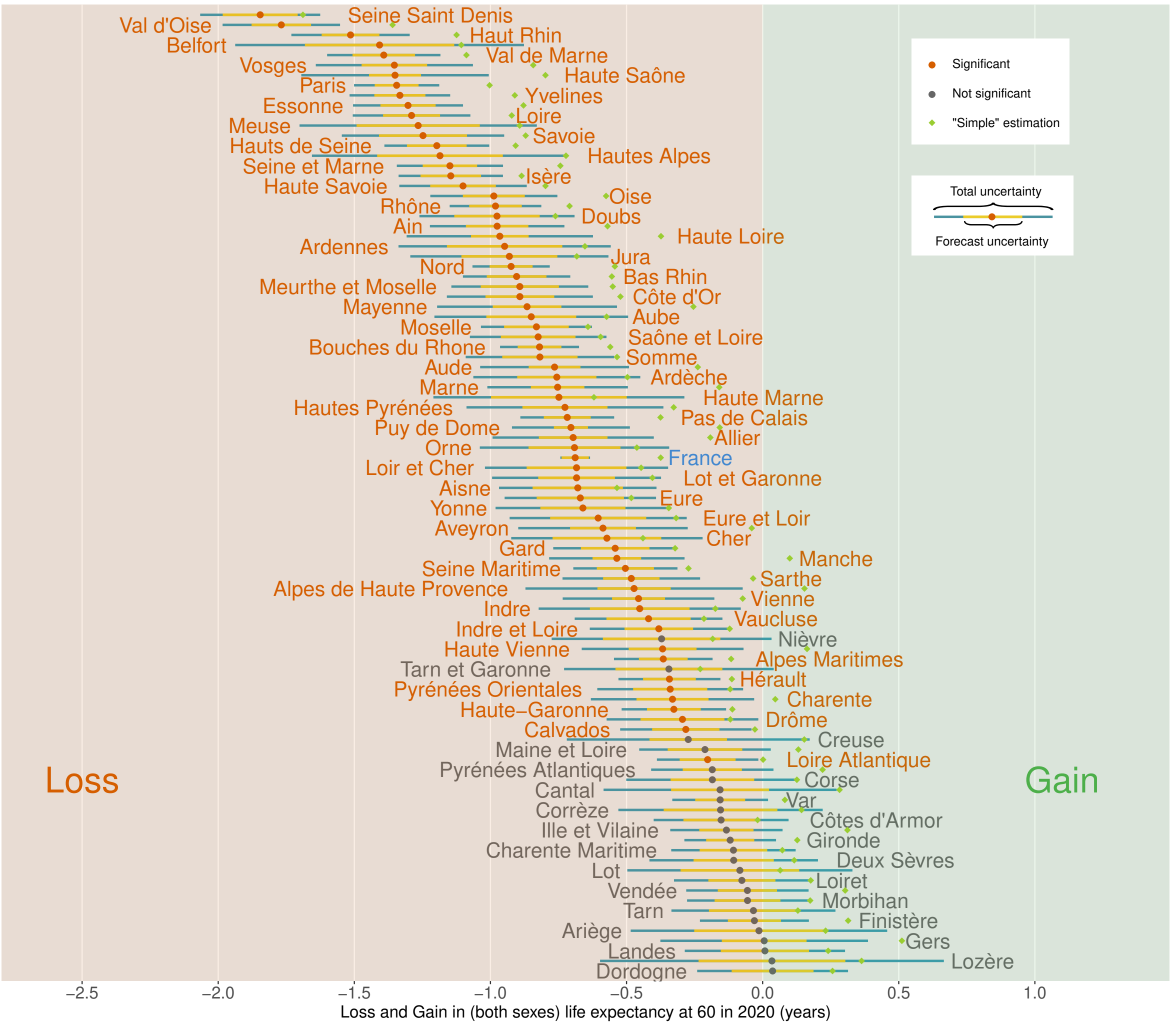

Figure 4: Losses in life expectancy at age 60 in 2020 for each French *département*, both sexes combined. Colors of dots and texts express the presence of significant estimates at 95% level, and colors of the horizontal bars represent the two sources of uncertainty. Green dots identify "simple" estimates of losses.

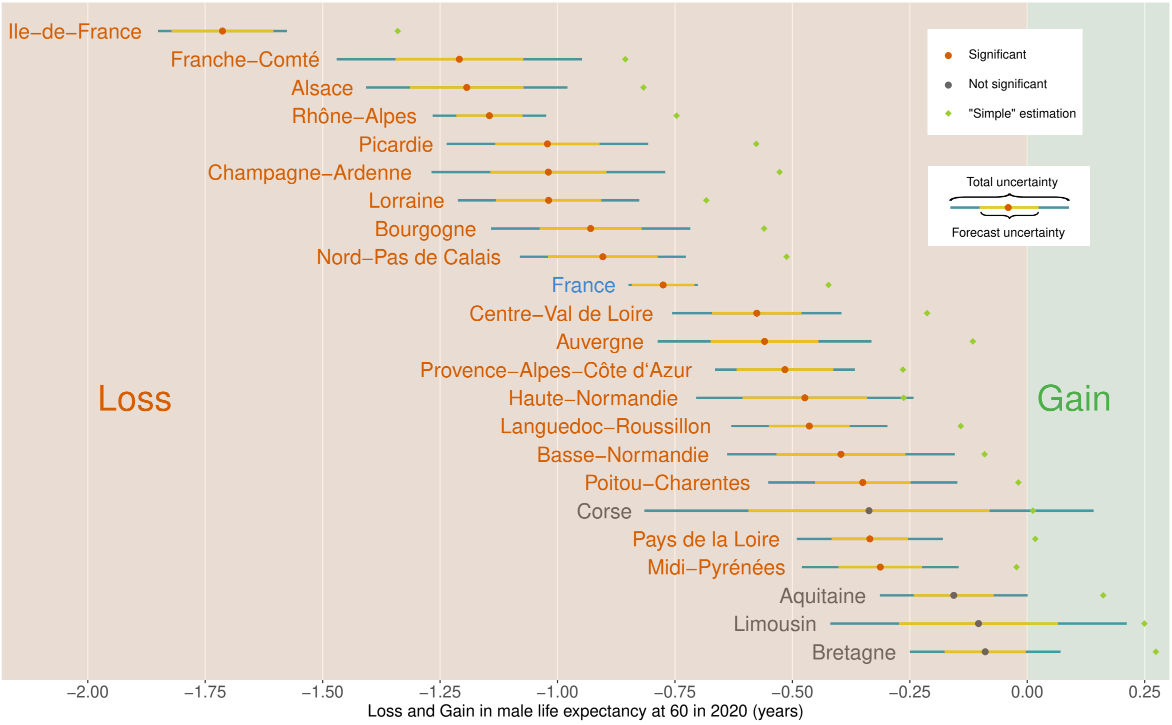

Figure 5: Losses in male life expectancy at age 60 in 2020 for each French *régions*. Colors of dots and texts express the presence of significant estimates at 95% level, and colors of the horizontal bars represent the two sources of uncertainty.

Table 1: Losses/gains in male life expectancy at age 60 in 2020 for each French *départements*, $\delta_{e_{60}}$, and associated 95% confidence interval (CI). "Simple" estimates for $\delta_{e_{60}}$ are also provided, as well as life expectancy at age 60 observed in 2015-2019 ($e_{60}^{2015-19}$ obs), life expectancy at age 60 observed in 2020 (e_{60}^{2020} obs) and life expectancy at age 60 forecast in 2020 (e_{60}^{2020} for).

Département	$e_{60}^{2015-19}$ obs	e_{60}^{2020} obs	e_{60}^{2020} for	Simple $\delta_{e_{60}}$	$\delta_{e_{60}}$	95% CI
Ain	23.65	23.02	24.1	-0.63	-1.08	[-1.44; -0.72]
Aisne	21.51	20.86	21.75	-0.65	-0.9	[-1.31; -0.49]
Allier	22.46	22.17	22.85	-0.29	-0.68	[-1.13; -0.23]
Alpes de Hte Pce	23.29	23.34	23.91	0.05	-0.57	[-1.15; 0]
Hautes Alpes	24.07	23.39	24.72	-0.68	-1.34	[-1.94; -0.73]
Alpes Maritimes	23.7	23.62	24.01	-0.08	-0.39	[-0.66; -0.12]
Ardèche	23.18	22.56	23.66	-0.62	-1.1	[-1.52; -0.67]
Ardennes	21.91	21.51	22.23	-0.4	-0.72	[-1.28; -0.16]
Ariège	23.25	23.45	23.75	0.2	-0.3	[-0.91; 0.31]
Aube	22.48	21.58	22.77	-0.9	-1.19	[-1.7; -0.67]
Aude	23.16	22.99	23.75	-0.17	-0.76	[-1.15; -0.37]
Aveyron	23.71	23.83	24.24	0.12	-0.42	[-0.86; 0.02]
Bouches du Rhone	23.46	22.86	23.9	-0.6	-1.04	[-1.24; -0.83]
Calvados	22.58	22.66	22.8	0.08	-0.14	[-0.5; 0.22]
Cantal	22.46	22.35	22.56	-0.11	-0.21	[-0.88; 0.45]
Charente	23.14	23.14	23.65	0	-0.52	[-0.92; -0.11]
Charente Maritime	23.19	23.18	23.45	-0.01	-0.26	[-0.59; 0.06]
Cher	22.23	21.62	22.74	-0.61	-1.12	[-1.55; -0.7]
Corrèze	23	23.13	23.27	0.13	-0.14	[-0.66; 0.38]
Corse	24.03	24.04	24.33	0.01	-0.29	[-0.77; 0.18]
Côte d'Or	23.53	22.71	23.97	-0.82	-1.26	[-1.62; -0.9]
Côtes d'Armor	22.51	22.49	22.73	-0.01	-0.23	[-0.59; 0.12]
Creuse	21.71	22.22	22.07	0.51	0.14	[-0.46; 0.75]
Dordogne	23.02	23.27	23.3	0.25	-0.03	[-0.43; 0.37]
Doubs	23.38	22.53	23.68	-0.85	-1.15	[-1.56; -0.73]
Drôme	23.52	23.18	23.69	-0.34	-0.51	[-0.92; -0.11]
Eure	22.19	21.8	22.63	-0.39	-0.84	[-1.19; -0.49]
Eure et Loir	22.76	22.33	22.98	-0.43	-0.65	[-1.13; -0.18]
Finistère	21.93	22.35	22.31	0.42	0.04	[-0.26; 0.34]
Gard	23.25	22.98	23.63	-0.27	-0.65	[-0.96; -0.33]
Haute-Garonne	24.03	23.97	24.21	-0.06	-0.24	[-0.53; 0.05]
Gers	23.47	24.21	23.62	0.73	0.59	[0; 1.17]
Gironde	23.46	23.69	23.69	0.23	0	[-0.25; 0.26]
Hérault	23.4	23.3	23.61	-0.11	-0.31	[-0.6; -0.02]
Ille et Vilaine	23.42	23.76	23.84	0.34	-0.08	[-0.38; 0.23]
Indre	22.22	22.07	22.7	-0.14	-0.62	[-1.12; -0.13]
Indre et Loire	23.7	23.66	24.06	-0.03	-0.4	[-0.76; -0.03]

Table 1 continued from previous page

Département	$e_{60}^{2015-19}$ obs	e_{60}^{2020} obs	e_{60}^{2020} for	Simple $\delta_{e_{60}}$	$\delta_{e_{60}}$	95% CI
Isère	23.96	23.04	24.28	-0.92	-1.24	[-1.52; -0.96]
Jura	23.24	22.75	23.5	-0.49	-0.75	[-1.28; -0.22]
Landes	23.24	23.51	23.55	0.27	-0.04	[-0.47; 0.39]
Loir et Cher	23.27	22.78	23.48	-0.48	-0.7	[-1.19; -0.2]
Loire	23.29	22.42	23.77	-0.87	-1.35	[-1.66; -1.03]
Haute Loire	22.49	22.4	22.93	-0.09	-0.53	[-1.02; -0.05]
Loire Atlantique	22.82	22.79	23.15	-0.03	-0.36	[-0.63; -0.09]
Loiret	23.11	23.27	23.41	0.16	-0.13	[-0.5; 0.24]
Lot	23.71	23.87	23.88	0.16	-0.01	[-0.62; 0.59]
Lot et Garonne	23.33	22.75	23.47	-0.58	-0.72	[-1.2; -0.24]
Lozère	22.17	22.85	22.52	0.68	0.33	[-0.56; 1.22]
Maine et Loire	23.94	23.94	24.36	0	-0.42	[-0.78; -0.06]
Manche	22.7	22.78	23.45	0.08	-0.67	[-1.03; -0.31]
Marne	22.18	21.89	22.84	-0.3	-0.96	[-1.33; -0.59]
Haute Marne	22.11	21.36	22.23	-0.75	-0.87	[-1.52; -0.22]
Mayenne	23.77	23.43	24.47	-0.35	-1.04	[-1.51; -0.57]
Meurthe et Moselle	22.6	22.09	23.06	-0.51	-0.97	[-1.31; -0.64]
Meuse	22.03	21.15	22.48	-0.88	-1.33	[-1.94; -0.71]
Morbihan	22.44	22.71	22.8	0.27	-0.08	[-0.39; 0.23]
Moselle	22.1	21.42	22.36	-0.68	-0.94	[-1.23; -0.65]
Nièvre	21.82	21.58	22.19	-0.24	-0.61	[-1.12; -0.11]
Nord	21.33	20.79	21.67	-0.54	-0.88	[-1.1; -0.66]
Oise	22.19	21.56	22.68	-0.62	-1.12	[-1.44; -0.79]
Orne	23	22.29	23.3	-0.71	-1.01	[-1.51; -0.51]
Pas de Calais	20.77	20.31	21.13	-0.47	-0.82	[-1.07; -0.57]
Puy de Dome	22.91	22.89	23.37	-0.02	-0.48	[-0.8; -0.16]
Pyrénées Atlantiques	23.49	23.78	23.88	0.29	-0.1	[-0.43; 0.23]
Hautes Pyrénées	23.26	22.7	23.65	-0.56	-0.95	[-1.49; -0.42]
Pyrénées Orientales	22.95	22.77	23.22	-0.18	-0.45	[-0.85; -0.04]
Bas Rhin	22.91	22.49	23.28	-0.42	-0.8	[-1.08; -0.51]
Haut Rhin	23.03	21.69	23.51	-1.34	-1.82	[-2.13; -1.51]
Rhône	24.04	23.32	24.38	-0.72	-1.06	[-1.3; -0.81]
Haute Saône	22.68	21.71	23.09	-0.97	-1.37	[-1.91; -0.83]
Saône et Loire	23.1	22.52	23.36	-0.57	-0.84	[-1.2; -0.47]
Sarthe	23.32	23.25	23.8	-0.07	-0.55	[-0.92; -0.18]
Savoie	23.73	22.8	24.15	-0.94	-1.35	[-1.78; -0.93]
Haute Savoie	23.82	23.05	24.2	-0.77	-1.15	[-1.49; -0.81]
Seine Maritime	22.09	21.89	22.32	-0.2	-0.43	[-0.71; -0.15]
Seine et Marne	23.1	22.08	23.48	-1.02	-1.39	[-1.69; -1.1]
Deux Sèvres	23.53	23.46	23.8	-0.07	-0.34	[-0.8; 0.11]
Somme	21.65	21.18	21.97	-0.47	-0.79	[-1.18; -0.4]
Tarn	24	23.86	24.21	-0.15	-0.35	[-0.78; 0.08]
Tarn et Garonne	23.39	23.12	23.97	-0.27	-0.85	[-1.35; -0.34]
Var	23.45	23.48	23.66	0.03	-0.18	[-0.45; 0.09]

Table 1 continued from previous page

Département	$e_{60}^{2015-19}$ obs	e_{60}^{2020} obs	e_{60}^{2020} for	Simple $\delta_{e_{60}}$	$\delta_{e_{60}}$	95% CI
Vaucluse	23.14	22.99	23.43	-0.16	-0.44	[-0.83; -0.05]
Vendée	22.94	23.27	23.08	0.32	0.19	[-0.15; 0.53]
Vienne	23.51	23.51	23.87	0	-0.36	[-0.76; 0.04]
Haute Vienne	23.12	23.35	23.62	0.23	-0.26	[-0.7; 0.18]
Vosges	22.17	21.33	22.72	-0.84	-1.39	[-1.79; -0.98]
Yonne	22.08	21.59	22.39	-0.48	-0.8	[-1.27; -0.33]
Belfort	22.8	21.41	23.2	-1.39	-1.79	[-2.49; -1.1]
Essonne	24.05	23.13	24.41	-0.92	-1.28	[-1.58; -0.99]
Hauts de Seine	24.62	23.39	24.98	-1.23	-1.59	[-1.88; -1.31]
Seine Saint Denis	23.13	20.94	23.34	-2.19	-2.4	[-2.7; -2.1]
Val de Marne	24.02	22.7	24.34	-1.31	-1.64	[-1.96; -1.32]
Val d'Oise	23.25	21.71	23.79	-1.55	-2.09	[-2.39; -1.78]
Paris	24.63	23.36	24.94	-1.26	-1.57	[-1.86; -1.28]
Yvelines	24.51	23.37	25.01	-1.13	-1.64	[-1.9; -1.37]

Table 2: Losses/gains in female life expectancy at age 60 in 2020 for each French *départements*, $\delta_{e_{60}}$, and associated 95% confidence interval (CI). "Simple" estimates for $\delta_{e_{60}}$ are also provided, as well as life expectancy at age 60 observed in 2015-2019 ($e_{60}^{2015-19}$ obs), life expectancy at age 60 observed in 2020 (e_{60}^{2020} obs) and life expectancy at age 60 forecast in 2020 (e_{60}^{2020} for).

Département	$e_{60}^{2015-19}$ obs	e_{60}^{2020} obs	e_{60}^{2020} for	Simple $\delta_{e_{60}}$	$\delta_{e_{60}}$	95% CI
Ain	27.51	27.05	27.62	-0.46	-0.57	[-0.93; -0.22]
Aisne	25.81	25.48	25.81	-0.33	-0.33	[-0.72; 0.05]
Allier	27.05	27	27.46	-0.05	-0.45	[-0.82; -0.08]
Alpes de Hte Pce	27.12	27.4	27.86	0.28	-0.46	[-0.97; 0.06]
Hautes Alpes	27.96	27.21	28.23	-0.75	-1.02	[-1.65; -0.4]
Alpes Maritimes	27.56	27.43	27.86	-0.13	-0.44	[-0.66; -0.21]
Ardèche	27.06	26.75	27.25	-0.31	-0.51	[-0.91; -0.1]
Ardennes	26.22	25.34	26.5	-0.88	-1.16	[-1.65; -0.67]
Ariège	27.2	27.47	27.69	0.27	-0.22	[-0.79; 0.34]
Aube	26.92	26.78	27.24	-0.14	-0.45	[-0.9; 0]
Aude	27.25	26.94	27.52	-0.31	-0.58	[-0.97; -0.2]
Aveyron	27.63	27.43	28.06	-0.2	-0.63	[-1.04; -0.23]
Bouches du Rhone	27.32	26.85	27.55	-0.47	-0.7	[-0.88; -0.51]
Calvados	27.15	27.03	27.21	-0.12	-0.18	[-0.51; 0.15]
Cantal	26.59	27.38	26.95	0.79	0.43	[-0.13; 0.98]
Charente	27.21	27.35	27.13	0.13	0.22	[-0.22; 0.65]
Charente Maritime	27.45	27.61	27.73	0.17	-0.12	[-0.4; 0.16]
Cher	26.51	26.33	26.71	-0.18	-0.38	[-0.83; 0.07]
Corrèze	27.24	27.41	27.46	0.17	-0.05	[-0.53; 0.44]
Corse	27.57	27.82	28.01	0.25	-0.19	[-0.57; 0.19]
Côte d'Or	27.44	27.31	27.78	-0.13	-0.47	[-0.81; -0.12]
Côtes d'Armor	26.97	26.97	27.23	0	-0.26	[-0.56; 0.04]
Creuse	26.41	26.12	26.92	-0.29	-0.79	[-1.4; -0.18]
Dordogne	27.08	27.37	27.47	0.28	-0.1	[-0.43; 0.23]
Doubs	27.31	26.7	27.3	-0.61	-0.6	[-0.98; -0.22]
Drôme	27.46	27.6	27.52	0.14	0.08	[-0.29; 0.45]
Eure	26.64	26.1	26.76	-0.54	-0.66	[-1.02; -0.31]
Eure et Loir	27.06	26.88	27.33	-0.18	-0.45	[-0.87; -0.03]
Finistère	26.7	26.93	26.98	0.23	-0.05	[-0.3; 0.2]
Gard	27.31	26.96	27.54	-0.35	-0.58	[-0.86; -0.29]
Haute-Garonne	27.86	27.72	27.9	-0.14	-0.18	[-0.44; 0.08]
Gers	27.5	27.74	27.85	0.24	-0.11	[-0.67; 0.44]
Gironde	27.58	27.6	27.68	0.02	-0.08	[-0.31; 0.15]
Hérault	27.53	27.43	27.73	-0.1	-0.3	[-0.54; -0.07]
Ille et Vilaine	27.69	27.98	27.93	0.29	0.04	[-0.22; 0.31]
Indre	26.68	26.53	27.02	-0.15	-0.49	[-0.95; -0.04]
Indre et Loire	27.99	27.81	28.15	-0.18	-0.34	[-0.66; -0.02]

Table 2 continued from previous page

Département	$e_{60}^{2015-19}$ obs	e_{60}^{2020} obs	e_{60}^{2020} for	Simple $\delta_{e_{60}}$	$\delta_{e_{60}}$	95% CI
Isère	27.72	26.94	27.98	-0.78	-1.04	[-1.28; -0.8]
Jura	27.54	26.7	27.94	-0.84	-1.24	[-1.69; -0.78]
Landes	27.34	27.59	27.4	0.25	0.19	[-0.2; 0.58]
Loir et Cher	27.21	26.83	27.2	-0.38	-0.37	[-0.82; 0.09]
Loire	27.36	26.47	27.57	-0.88	-1.1	[-1.38; -0.81]
Haute Loire	27.1	26.45	27.55	-0.66	-1.1	[-1.56; -0.65]
Loire Atlantique	27.43	27.51	27.53	0.08	-0.02	[-0.26; 0.22]
Loiret	27.18	27.4	27.3	0.22	0.1	[-0.22; 0.42]
Lot	27.51	27.52	27.55	0.01	-0.03	[-0.58; 0.52]
Lot et Garonne	27.45	27.27	27.6	-0.18	-0.33	[-0.74; 0.08]
Lozère	26.86	26.82	27.17	-0.03	-0.34	[-1.17; 0.48]
Maine et Loire	28	28.3	28.24	0.29	0.06	[-0.25; 0.38]
Manche	27.35	27.52	27.54	0.16	-0.02	[-0.36; 0.32]
Marne	26.74	26.74	27.25	0	-0.51	[-0.83; -0.18]
Haute Marne	26.58	26.16	27	-0.42	-0.84	[-1.39; -0.28]
Mayenne	27.75	27.65	28.45	-0.1	-0.8	[-1.22; -0.38]
Meurthe et Moselle	26.68	26.15	26.91	-0.54	-0.76	[-1.09; -0.43]
Meuse	26.48	25.71	26.86	-0.77	-1.15	[-1.67; -0.62]
Morbihan	27.1	27.17	27.28	0.07	-0.11	[-0.4; 0.18]
Moselle	25.96	25.41	26.05	-0.54	-0.64	[-0.92; -0.36]
Nièvre	26.62	26.56	26.73	-0.06	-0.17	[-0.68; 0.34]
Nord	26.01	25.52	26.41	-0.49	-0.89	[-1.05; -0.72]
Oise	26.35	25.89	26.61	-0.46	-0.71	[-1.02; -0.41]
Orne	27.41	27.28	27.52	-0.13	-0.23	[-0.68; 0.22]
Pas de Calais	25.77	25.58	26.08	-0.19	-0.5	[-0.71; -0.28]
Puy de Dome	27.13	26.86	27.66	-0.27	-0.8	[-1.09; -0.51]
Pyrénées Atlantiques	27.57	27.76	27.89	0.18	-0.13	[-0.42; 0.15]
Hautes Pyrénées	27.31	27.3	27.61	-0.01	-0.31	[-0.78; 0.16]
Pyrénées Orientales	27.18	27.16	27.27	-0.03	-0.11	[-0.47; 0.25]
Bas Rhin	26.88	26.25	27.17	-0.63	-0.92	[-1.17; -0.67]
Haut Rhin	26.71	25.93	27.04	-0.78	-1.1	[-1.39; -0.82]
Rhône	27.98	27.33	28.14	-0.65	-0.81	[-1.03; -0.59]
Haute Saône	26.71	26.17	27.31	-0.54	-1.14	[-1.61; -0.68]
Saône et Loire	27.4	26.85	27.54	-0.55	-0.69	[-1.02; -0.36]
Sarthe	27.51	27.54	27.85	0.03	-0.31	[-0.64; 0.03]
Savoie	27.71	26.98	27.96	-0.73	-0.98	[-1.39; -0.57]
Haute Savoie	27.73	26.96	28.05	-0.76	-1.09	[-1.39; -0.79]
Seine Maritime	26.76	26.46	26.93	-0.3	-0.46	[-0.71; -0.21]
Seine et Marne	26.85	26.47	27.2	-0.38	-0.72	[-0.96; -0.49]
Deux Sèvres	27.46	27.8	27.56	0.34	0.24	[-0.16; 0.64]
Somme	26.22	25.64	26.38	-0.58	-0.74	[-1.09; -0.38]
Tarn	27.78	28.23	27.89	0.45	0.34	[-0.06; 0.73]
Tarn et Garonne	27.33	27.21	27.71	-0.12	-0.5	[-0.98; -0.03]
Var	27.49	27.64	27.64	0.15	0	[-0.22; 0.23]

Table 2 continued from previous page

Département	$e_{60}^{2015-19}$ obs	e_{60}^{2020} obs	e_{60}^{2020} for	Simple $\delta_{e_{60}}$	$\delta_{e_{60}}$	95% CI
Vaucluse	27.18	26.94	27.22	-0.24	-0.28	[-0.64; 0.08]
Vendée	27.52	27.81	27.82	0.29	-0.01	[-0.29; 0.28]
Vienne	27.94	27.8	28.04	-0.14	-0.23	[-0.62; 0.16]
Haute Vienne	27.6	27.69	28.1	0.09	-0.42	[-0.79; -0.05]
Vosges	26.62	25.85	27.17	-0.77	-1.32	[-1.69; -0.95]
Yonne	26.35	26.21	26.44	-0.14	-0.23	[-0.67; 0.22]
Belfort	26.66	25.97	26.98	-0.69	-1	[-1.66; -0.35]
Essonne	27.69	26.91	27.98	-0.78	-1.07	[-1.35; -0.8]
Hauts de Seine	27.96	27.41	28.13	-0.55	-0.71	[-0.96; -0.46]
Seine Saint Denis	26.67	25.61	27.09	-1.06	-1.48	[-1.74; -1.21]
Val de Marne	27.81	27.02	28	-0.79	-0.99	[-1.26; -0.72]
Val d'Oise	27.01	25.94	27.19	-1.07	-1.25	[-1.55; -0.94]
Paris	28.2	27.52	28.5	-0.68	-0.98	[-1.18; -0.78]
Yvelines	28.04	27.41	28.4	-0.63	-0.99	[-1.23; -0.76]

Table 3: Losses/gains in both sexes life expectancy at age 60 in 2020 for each French *départements*, $\delta_{e_{60}}$, and associated 95% confidence interval (CI). "Simple" estimates for $\delta_{e_{60}}$ are also provided, as well as life expectancy at age 60 observed in 2015-2019 ($e_{60}^{2015-19}$ obs), life expectancy at age 60 observed in 2020 (e_{60}^{2020} obs) and life expectancy at age 60 forecast in 2020 (e_{60}^{2020} for).

Département	$e_{60}^{2015-19}$ obs	e_{60}^{2020} obs	e_{60}^{2020} for	Simple $\delta_{e_{60}}$	$\delta_{e_{60}}$	95% CI
Ain	25.64	25.08	26.05	-0.57	-0.98	[-1.22; -0.73]
Aisne	23.74	23.21	23.88	-0.54	-0.68	[-0.97; -0.39]
Allier	24.86	24.67	25.37	-0.19	-0.7	[-0.99; -0.4]
Alpes de Hte Pce	25.24	25.4	25.87	0.15	-0.47	[-0.87; -0.07]
Hautes Alpes	26.07	25.35	26.53	-0.72	-1.19	[-1.66; -0.71]
Alpes Maritimes	25.78	25.66	26.03	-0.12	-0.37	[-0.55; -0.18]
Ardèche	25.17	24.68	25.43	-0.5	-0.76	[-1.06; -0.45]
Ardennes	24.16	23.5	24.45	-0.65	-0.95	[-1.34; -0.56]
Ariège	25.25	25.48	25.49	0.23	-0.01	[-0.48; 0.46]
Aube	24.81	24.24	25.09	-0.57	-0.85	[-1.21; -0.49]
Aude	25.26	25.02	25.79	-0.24	-0.76	[-1.04; -0.49]
Aveyron	25.71	25.67	26.26	-0.04	-0.59	[-0.9; -0.28]
Bouches du Rhone	25.52	24.96	25.78	-0.56	-0.82	[-0.96; -0.67]
Calvados	25.01	24.98	25.27	-0.03	-0.28	[-0.52; -0.04]
Cantal	24.56	24.84	25	0.28	-0.16	[-0.58; 0.27]
Charente	25.25	25.3	25.63	0.05	-0.33	[-0.63; -0.03]
Charente Maritime	25.42	25.49	25.6	0.07	-0.11	[-0.34; 0.12]
Cher	24.43	23.99	24.56	-0.44	-0.57	[-0.92; -0.22]
Corrèze	25.22	25.37	25.52	0.14	-0.15	[-0.53; 0.22]
Corse	25.85	25.97	26.16	0.13	-0.18	[-0.5; 0.13]
Côte d'Or	25.59	25.07	25.96	-0.52	-0.89	[-1.16; -0.62]
Côtes d'Armor	24.83	24.82	24.97	-0.02	-0.15	[-0.4; 0.1]
Creuse	24.07	24.23	24.5	0.15	-0.27	[-0.72; 0.17]
Dordogne	25.11	25.36	25.33	0.26	0.04	[-0.24; 0.31]
Doubs	25.44	24.68	25.65	-0.76	-0.98	[-1.26; -0.69]
Drôme	25.59	25.47	25.76	-0.12	-0.29	[-0.57; -0.02]
Eure	24.49	24.01	24.68	-0.48	-0.67	[-0.95; -0.39]
Eure et Loir	24.96	24.64	25.25	-0.32	-0.6	[-0.93; -0.28]
Finistère	24.45	24.76	24.79	0.31	-0.03	[-0.23; 0.17]
Gard	25.36	25.04	25.58	-0.32	-0.54	[-0.77; -0.31]
Haute-Garonne	26.08	25.96	26.29	-0.11	-0.33	[-0.52; -0.13]
Gers	25.51	26.02	26.01	0.51	0.01	[-0.38; 0.39]
Gironde	25.66	25.79	25.91	0.13	-0.12	[-0.29; 0.05]
Hérault	25.57	25.46	25.8	-0.11	-0.34	[-0.53; -0.16]
Ille et Vilaine	25.69	26	26.13	0.31	-0.13	[-0.34; 0.07]
Indre	24.5	24.33	24.78	-0.17	-0.45	[-0.82; -0.08]
Indre et Loire	25.96	25.84	26.22	-0.12	-0.38	[-0.64; -0.13]

Table 3 continued from previous page

Département	$e_{60}^{2015-19}$ obs	e_{60}^{2020} obs	e_{60}^{2020} for	Simple $\delta_{e_{60}}$	$\delta_{e_{60}}$	95% CI
Isère	25.95	25.06	26.21	-0.89	-1.15	[-1.34; -0.95]
Jura	25.47	24.78	25.72	-0.68	-0.93	[-1.29; -0.57]
Landes	25.37	25.62	25.61	0.24	0.01	[-0.29; 0.3]
Loir et Cher	25.31	24.86	25.54	-0.45	-0.68	[-1.02; -0.35]
Loire	25.46	24.54	25.83	-0.92	-1.29	[-1.51; -1.07]
Haute Loire	24.85	24.48	25.44	-0.37	-0.97	[-1.31; -0.62]
Loire Atlantique	25.28	25.28	25.48	0	-0.2	[-0.39; -0.02]
Loiret	25.23	25.41	25.49	0.18	-0.08	[-0.33; 0.17]
Lot	25.66	25.73	25.81	0.06	-0.08	[-0.5; 0.33]
Lot et Garonne	25.46	25.05	25.74	-0.4	-0.68	[-0.99; -0.37]
Lozère	24.47	24.83	24.8	0.36	0.03	[-0.6; 0.67]
Maine et Loire	26.07	26.2	26.41	0.13	-0.21	[-0.45; 0.03]
Manche	25.1	25.2	25.73	0.1	-0.54	[-0.78; -0.29]
Marne	24.59	24.43	25.18	-0.16	-0.75	[-1.01; -0.5]
Haute Marne	24.41	23.79	24.54	-0.62	-0.75	[-1.21; -0.29]
Mayenne	25.83	25.58	26.45	-0.25	-0.87	[-1.2; -0.54]
Meurthe et Moselle	24.78	24.23	25.12	-0.55	-0.89	[-1.14; -0.64]
Meuse	24.32	23.43	24.69	-0.89	-1.27	[-1.7; -0.83]
Morbihan	24.87	25.05	25.1	0.17	-0.06	[-0.28; 0.17]
Moselle	24.13	23.48	24.32	-0.64	-0.83	[-1.03; -0.63]
Nièvre	24.3	24.12	24.49	-0.18	-0.37	[-0.78; 0.03]
Nord	23.83	23.29	24.21	-0.54	-0.92	[-1.07; -0.78]
Oise	24.35	23.78	24.77	-0.58	-0.99	[-1.22; -0.75]
Orne	25.27	24.81	25.5	-0.46	-0.69	[-1.04; -0.34]
Pas de Calais	23.43	23.06	23.78	-0.38	-0.72	[-0.89; -0.55]
Puy de Dome	25.14	24.99	25.69	-0.16	-0.7	[-0.92; -0.49]
Pyrénées Atlantiques	25.66	25.88	26.07	0.22	-0.18	[-0.41; 0.04]
Hautes Pyrénées	25.4	25.07	25.8	-0.33	-0.73	[-1.09; -0.36]
Pyrénées Orientales	25.16	25.05	25.38	-0.12	-0.34	[-0.61; -0.07]
Bas Rhin	24.99	24.44	25.34	-0.55	-0.9	[-1.1; -0.71]
Haut Rhin	24.95	23.82	25.34	-1.12	-1.51	[-1.73; -1.3]
Rhône	26.17	25.46	26.45	-0.71	-0.98	[-1.15; -0.81]
Haute Saône	24.74	23.94	25.29	-0.8	-1.35	[-1.69; -1.01]
Saône et Loire	25.33	24.73	25.56	-0.6	-0.82	[-1.08; -0.57]
Sarthe	25.5	25.47	25.95	-0.04	-0.48	[-0.74; -0.23]
Savoie	25.83	24.96	26.21	-0.87	-1.25	[-1.55; -0.95]
Haute Savoie	25.9	25.1	26.2	-0.8	-1.1	[-1.33; -0.87]
Seine Maritime	24.57	24.3	24.8	-0.27	-0.5	[-0.7; -0.31]
Seine et Marne	25.08	24.34	25.49	-0.74	-1.15	[-1.34; -0.95]
Deux Sèvres	25.55	25.67	25.78	0.12	-0.11	[-0.42; 0.2]
Somme	24.03	23.49	24.31	-0.53	-0.82	[-1.09; -0.55]
Tarn	25.98	26.11	26.15	0.13	-0.03	[-0.34; 0.27]
Tarn et Garonne	25.42	25.19	25.54	-0.23	-0.34	[-0.73; 0.04]
Var	25.56	25.64	25.8	0.08	-0.16	[-0.33; 0.02]

Table 3 continued from previous page

Département	$e_{60}^{2015-19}$ obs	e_{60}^{2020} obs	e_{60}^{2020} for	Simple $\delta_{e_{60}}$	$\delta_{e_{60}}$	95% CI
Vaucluse	25.26	25.04	25.46	-0.22	-0.42	[-0.69; -0.15]
Vendée	25.3	25.6	25.65	0.3	-0.06	[-0.28; 0.17]
Vienne	25.81	25.73	26.19	-0.07	-0.46	[-0.73; -0.18]
Haute Vienne	25.47	25.63	26	0.16	-0.37	[-0.66; -0.07]
Vosges	24.49	23.65	25	-0.84	-1.35	[-1.64; -1.06]
Yonne	24.27	23.92	24.58	-0.34	-0.66	[-0.98; -0.34]
Belfort	24.85	23.74	25.15	-1.11	-1.41	[-1.94; -0.88]
Essonne	25.98	25.1	26.4	-0.88	-1.3	[-1.5; -1.1]
Hauts de Seine	26.48	25.57	26.77	-0.91	-1.2	[-1.39; -1]
Seine Saint Denis	24.98	23.29	25.13	-1.69	-1.85	[-2.07; -1.63]
Val de Marne	26.09	25	26.39	-1.09	-1.39	[-1.6; -1.18]
Val d'Oise	25.24	23.88	25.65	-1.36	-1.77	[-1.98; -1.55]
Paris	26.62	25.62	26.96	-1	-1.34	[-1.5; -1.19]
Yvelines	26.4	25.49	26.82	-0.91	-1.33	[-1.52; -1.15]
