# Supplementary material for: Estimating subnational excess mortality in times of pandemic. An application to French départements in 2020
Source: PLoS One. 2024 Jan 19;19(1):e0293752. doi: 10.1371/journal.pone.0293752 (PMC10798530; doi:10.1371/journal.pone.0293752)
Supplement: Attachment — Submitted filename: Response to Reviewers.pdf [file pone.0293752.s004.pdf]

Rebuttal letter for

Estimating Subnational Excess Mortality in Times of Pandemic. An application to French départements in 2020

Submitted to PLOS One

PONE-D-23-16951

July 31, 2023

Two small notes:

- Some questions were common to more reviewers and, in replying, we followed the order as in PLOS One Decision letter. Consequently and for some comments, we could have been more detailed in responding to Reviewer #1. However, we always referenced to other responses when necessary.
- To facilitate the identification of modifications made from the previous draft, we have highlighted alterations to the manuscript using the color red. However, it's worth noting that simple language changes resulting from the English editing are not marked. To enhance readability and following PLOS One requests, we also enclose a revised version of the paper that does not reference any specific modifications made.

We received no specific funding for this work.

Academic Editor

1) Please ensure that your manuscript meets PLOS ONE's style requirements, including those for file naming.

> Our manuscript meets PLOS ONE's style requirements.

2) Please note that PLOS ONE has specific guidelines on code sharing for submissions in which author-generated code underpins the findings in the manuscript. In these cases, all author-generated code must be made available without restrictions upon publication of the work.

> The code and data needed to replicate our results are unlimitedly available and can be accessed through this link:
> https://osf.io/zt2c8/?view_only=16ff2a7384c04659bdc39c6a223f2403
> (currently made anonymous for peer-review reasons).

3) Thank you for stating the following financial disclosure: [...] Please include your amended statements within your cover letter; we will change the online submission form on your behalf.

> We have added a sentence in the cover letter.

4) We note that Figure 3 in your submission contain [map/satellite] images which may be copyrighted. All PLOS content is published under the Creative Commons Attribution License (CC BY 4.0) [...]

> Figure 3 was produced using a dedicated application created by CNRS (French National Institute for Scientific Research), based on fully open-source code and resources.
> https://magrit.cnrs.fr/
> Therefore, our figure 3 can be published under the Creative Commons Attribution License (CC BY 4.0).

5) I have received 3 reports from 3 referees. They are raising some issues that you have to deal with, before resubmitting. Please, have a scrupulous attention on these 3 reports below.

> We are very grateful to the 3 referees for taking the time to study our manuscript thoroughly. Our responses follow each of their comments.

Reviewer #1

1) Even though the epidemic is over, the issue of the losses caused by it, in particular its detailed analysis, remains important. The paper does a very good job of explaining the regional variation in excess mortality during the COVID period in France and can make a very good contribution to the field.

   The general approach of the paper (based on full mortality projections rather than base year figures or simplified extrapolations of deaths) is sound, the text is well written.

   We would like to thank you sincerely for your kind comments about our research.

2) However, my main concern is with the CP-splines used in the paper. Apart from general concerns about splines used to extrapolate beyond the data to which they are fitted, the extrapolations by the CP-splines are partly based on ad hoc solutions, such as limiting the rate of change in mortality to 50% of its variation in the past. This could, for example, lead to overly pessimistic projections of mortality decline. It could also reduce estimates of the uncertainty of the prediction (in what I read about the method, I did not see discussion of whether the ad hoc constraints imposed on the model might affect the uncertainty; please check this point). I suggest checking the validity of CP-splines based extrapolations by comparing them, at least in some cases, with a simpler, more transparent method, such as linear extrapolation of log-mortality rates, or the technically similar Lee-Carter method. If the methods appear to give similar results in terms of excess mortality for selected regions, this would be a good support for your methodology, especially for those readers for whom the CP-splines are too technical a model. In any case, you might need to explain the basics of CP-splines and their use for extrapolation in more detail, but in simple language; discussing possible limitations of the method and its estimated uncertainty.

   We appreciate you bringing up these concerns and providing us with the opportunity to address these important concepts.

   CP-splines, an advanced variation of penalized splines (Eilers and Marx, 1996), go beyond merely extrapolating a single time-series by splines. Instead, they embed the entire age-time mortality development within a system of equations in which (the Kronecker product of) B-splines serve as regression bases. Associated coefficients are estimated and smooth by discrete and anisotropic penalty. To achieve forecasts, zero regression weights are assumed for future years (Currie et al., 2004). The disadvantage of this last approach was its failure to consider observed counts within a demographic framework.

   On the contrary, the CP-splines employed in this paper incorporate demographic information effectively. They enforce future age-patterns and age-specific rate-of-change to lie within a range of possible shapes and patterns computed from historical data, resulting in a more robust and contextually informed modeling approach.

As you rightly pointed out, certain choices must also be made when using CP-splines. For instance, in our case, we opted to enforce the future age-specific rate-of-change within the 50% confidence interval of the past age-specific rate-of-change. It's essential to emphasize that this choice is not an ad hoc solution, but rather a explicit choice that users need to be made. In introducing CP-splines, Camarda (2019) proposed this percentage based on extensive experiments, but forecasters are free to change based on their hypotheses and expertise.

In the forecasting literature, as you pointed out, an alternative is the widely spread Lee-Carter model (Lee and Carter, 1992) and its numerous variants. When this model is employed no much choice can be made. In the model aspect we discuss, future age-specific rate-of-change in the Lee-Carter will be *exactly equal* to the mean observed age-specific rate-of-change measured from the observed mortality. Moreover additional assumptions such as the linearity of the time index are intrinsically acquired when the Lee-Carter model is employed. Therefore it is undeniable that the Lee-Carter model is simpler. However, we hold a differing opinion regarding its transparency: few forecasters truly acknowledge that by using the Lee-Carter model, they enforce a fixed rate-of-improvement for each age in the future mortality.

In conclusions, opting for a 50% confidence interval for the rate-of-change in CP-splines grants greater flexibility to the future mortality development, making it particularly suitable for modeling small spatial areas. At the same time, the enforced smoothness achieved with penalized B-splines ensures a level of robustness that the Lee-Carter model lacks.

Based on your feedback, we have decided to revise the Supporting Information by conducting a comparison between CP-splines and the Lee-Carter model. In the current version, we focus on comparing excess life expectancy at age 60 for males using both approaches for all départements. We did not observe significant differences, especially for larger spatial units.

Following your suggestion for comparing the forecasting models "at least in some cases", we have selected the four *départements* with the most notable differences in excess mortality between the two approaches. For these units, we present trends in e_{60} and age-patterns in 2020, along with the estimated values for both the Lee-Carter and CP-splines methods. As described in the Supplementary Information, several aspects allows us to appreciate the advantages of CP-splines in modeling and forecasting mortality trends.

Given this new Section in the Supporting Information, we edited the main text accordingly:

"Additionally, Section B of the Supporting Information includes a comparison between the results obtained from the CP-splines and the more conventional Lee-Carter model (Lee and Carter, 1992). This additional analysis provides reassurance regarding the robustness of the majority of the outcomes. When significant discrepancies between the two models were observed in estimating excess mortality, the CP-splines demonstrated superior performance in both modeling and forecasting mortality in

small geographical units." (p. 5, l. 167)

We believe that our revised Supporting Information and additional information in the main manuscript will address any concerns you and PLOS One readers may have regarding the selection of the forecasting model used to calculate excess mortality in small geographical areas.

3) Finally, the discussion is too weak for such a topical paper as yours. I suggest that you provide a more detailed substantive discussion of what might explain the regional differences and perhaps put your results in the context of previous publications for France and other countries.

Thank you for your comment. We have tried to provide a more in-depth analysis of the results by comparing them with those produced by Institut national de la statistique et des études économiques (INSEE, the national statistics bureau of France) and analyzing them in the light of the two waves that affected the country. Here the paragraph we added in the manuscript:

"These results are consistent with those from the French national statistical office (INSEE) (Allard et al., 2020; Le Minez and Roux, 2021), which compared deaths observed in 2020 during the first two waves of the pandemic (March–April 2020 and September–December 2020) with those observed during the same periods in 2019. The first wave of the pandemic had strongly affected Greater Paris, as well as the north and northeast of the country, probably due to the onset of the first outbreaks in mainland France in these regions. One of the first outbreaks was detected in the Oise département, *in the immediate surroundings of the Charles de Gaulle airport (23 km northeast of Paris). Another outbreak was detected in* Haut-Rhin *and spread to the rest of northeastern France. The INSEE studies also showed that the second wave of the pandemic was strong throughout eastern France and particularly severe in the* Auvergne-Rhône-Alpes *region and neighboring* départements*."* (p. 6, l. 238)

Reviewer #2

1) This paper presents a method for estimating excess pandemic mortality on a sub-national scale. The authors present an application of their method to Covid-19 mortality data in France, at *département* level, in order to estimate excess Covid mortality in 2020. The paper is well produced overall, but a few imprecisions persist in the methodology presented and in some of the results, which would benefit from clarification.

   We would like to thank you sincerely for your constructive comments regarding our paper.

2) Section Methods, page 2, line 67: "However, without loss of generality, computation of excess mortality in successive years can be obtained by extending the forecast horizon presented below."

   It seems to me that by extending the forecasting period in this way, the estimates for the following years (2021+) would be somewhat deteriorated, as the French government's reaction to covid has not been constant throughout this period (mainly in 2020: containment and closure, in 2021: health pass and vaccine) and this may have a direct effect on mortality. The authors should therefore present the results at least for the year 2021, discussing both the quality of the forecasting and giving some hints of interpretation in terms of public policy and health context (appearance of new waves of variants).

   Thank you for bringing attention to this potentially misleading sentence in our paper. When calculating excess mortality, two critical factors come into play: the baseline mortality, which represents what would have been experienced in the absence of the pandemic, and the observed mortality levels during the pandemic. Rather than using the pre-pandemic mortality level as the baseline, we opted to estimate the baseline by forecasting pre-pandemic trends during the years of the pandemic. This approach enables us to directly consider the temporal changes in mortality, which may vary significantly across different subnational areas.

   Specifically, in our manuscript, we have placed a strong emphasis on all the methodological aspects that one needs to consider when measuring excess mortality at the subnational level, particularly focusing on uncertainty quantification.

   Regarding the sentence you referred to, our intention was to convey that when measuring excess mortality in, for instance, 2021, one would need to forecast pre-pandemic mortality two years ahead and have access to observed mortality data for the year 2021.

   We are fully aware that specific non-pharmaceutical interventions, as well as the implementation of vaccination campaigns during the course of the pandemic, could potentially affect mortality rates and, consequently, measures of excess mortality. However, these effects would be captured by the difference between the baseline and observed mortality, which is the fundamental definition of excess mortality. In other words, if containment

measures, closures, or vaccines had an impact, it would be evident when comparing the baseline mortality obtained by forecasting pre-pandemic mortality for the years affected by the pandemic.

To clarify these concepts and prevent any misleading interpretations, we have now edited the sentence to read as follows:

"However, the baseline mortality in the absence of the pandemic for successive years can be estimated by extending the forecast horizon of the model illustrated below. As a result, if subnational level data are accessible for 2021 and 2022, measuring excess mortality and quantify its uncertainty for these years can be achieved by simply adapting our approach." (p. 3, l. 79)

3) Section Methods, page 2, line 67: "For a given subpopulation and sex".

The authors should clarify early in the paper (also in the abstract) that the aim is to obtain a separate excess mortality estimator for men and women in each *département*. Furthermore, why differentiate between subpopulation and gender? While gender is a discriminating criterion, this needs to be clarified.

Furthermore, all the results presented are limited to the excess mortality in men, why this choice? It seems to me that this is not discussed in the paper; the idea of combining men and women to obtain less uncertainty is well discussed, as is the heterogeneity in mortality between men and women. The authors should give more reasons for this choice.

Thank you for your comment. We clarified in the abstract as well as in the introduction (p. 2, l. 61) and results part (p. 5, l. 179), pointing out that we estimate excess mortality for men and women independently, as well as for both sexes combined.

Furthermore, in this article we have emphasized the methodological aspects of estimating COVID-19-related excess mortality in small areas. In our first graphs, we have focused on males results, as they were more severely affected by the pandemic, while presenting the results for females in Supporting Information. However, in line with your comment, we have decided to replicate Figure 2 and 3 for females and provide these figures in the Supporting Information.

4) Section Methods, page 3, line 73: "Number of deaths d_{ij} at age i in year j are assumed to be realizations from a Poisson distribution"

Authors should add a sentence about the exposure n_{ij}, does it corresponds to the number of individuals at age i in year j ?

In the revised version of the manuscript, we have chosen to introduce the definition of exposures ahead of its previous placement in the Results section. Furthermore, we explicitly define exposures and provide the specific approximation used in our analysis.

"We define exposures ($\boldsymbol{N}$) as the mean of populations at 1st January for two consecutive years which is a suitable approximation of population exposed to the risk of death during a single age-time interval (Preston et al., 2001, p. 15) " (p. 3, l. 86)

5) Section Methods, page 3, line 84: "where $\boldsymbol{B}$ a two-dimensional model matrix that combines B-splines over age and years."

Although the paper makes a reference to "Camarda CG. Smooth Constrained Mortality Forecasting. Demographic Research. 2019;41(38):1091–1130. doi:10.4054/DemRes.2019.41.38." authors should give more insight about the way of computing this matrix and its relations with vectors $\boldsymbol{d}$ and $\boldsymbol{n}$, or maybe add a supplementary mathematical section.

We acknowledge that our description of the statistical aspects behind our approach may have been somewhat concise, particularly in the paragraph you mentioned. In light of your feedback, we have taken your suggestion and made the decision to incorporate more detailed insights and additional references in the Methods section to offer a more comprehensive explanation of our methodology.

"In simple terms, $\mathbf{B}$ is the result of a (Kronecker) product of two sets of equidistant B-splines, built separately over age and time. This construction gives rise to a dense pattern resembling an "egg carton," where each hill has an associated coefficient. The estimation process follows a classic regression Poisson setting, but with an additional discrete penalty to ensure smoothness of the coefficient vector $\boldsymbol{\alpha}$ and, consequently, smoothness of mortality $\boldsymbol{\mu}$ (Eilers and Marx, 1996). In addition to this framework, CP-splines impose constraints on $\boldsymbol{\alpha}$ for future years to align future 2020 mortality within shapes estimated from pre-pandemic years. Additional information regarding two-dimensional penalized B-splines can be referenced in Currie et al. (2004), while a comprehensive description of CP-splines is provided in Camarda (2019)." (p. 3, l. 100)

6) Section Methods, page 3, line 98: What is e_{2020} ? The denominator of the observed death rate ?
Section Methods, page 3, equation 2: The notation "e" is used here to represent the life expectancy at a given age, is it the same "e" referring to my previous question ? This should be clarified.

We sincerely apologize for this mistake. We have changed the text and replaced e_{2020} by n_{2020} (exposures) in the sentence.

"Whereas the former is obtained by CP-splines and it is solely dependent on the estimated coefficients $\boldsymbol{\alpha}$ in (1), observed death rates $\boldsymbol{m}_{2020} = \boldsymbol{d}_{2020}/\boldsymbol{n}_{2020}$ are the bases for computing actual level of mortality in the pandemic year." (p. 3, l. 121)

7) Figure 2 & 3:

   Looking at the map of French departments, there doesn't seem to be any significant gain, but figure 2 seems to say the opposite for the "Gers" department. Why does this department appear significant in figure 2 when it seems to be the same indicator shown in figure 3?

   We apologize for the confusion caused by a rounding error. The *département* of *Gers* did not enjoyed a significant gain in 2020. In Figure 2, the point estimate is now colored with grey (and not with orange); this color is used for non significant gain/loss. In Figure 2, "/" symbol is also used for non significant gain. Finally, in Table C.1, 0 is within the 95% confidence interval.

8) Overall, the article has a strong public health interest. Although there are still a few details to be taken into account and clarified, it informs and documents the literature on excess mortality in times of pandemic, with a well-executed application to the French *départements*.

   Many thanks for these kind comments.

Reviewer #3

1) This study estimates the loss of life expectancy in 2020 in 95 French departments. The study has qualities that are unfortunately masked by the fact that the paper is poorly written and structured. The paper's qualities are: 1) to give a picture of the sub-regional distribution of the impact of the pandemic in France 2020, 2) to use a sound method (already published) to project pre-pandemic mortality in 2020, and 3) to express results in terms of life expectancy, an important and intuitive metric. Unfortunately, the article in its current form puts too much emphasis on the method used, with too little comment on the results themselves, treated as a kind of "example" of applying the methodology rather than being the main result of the article. In addition, the writing in English is very poor and needs to be reviewed throughout the paper. The article should therefore be reworked to focus more on the results and less on the method (which is the subject of an earlier publication) and proofread by a native English speaker.

We would like to thank you sincerely for your helpful comments. Additionally, the paper underwent professional English editing to enhance its clarity and coherence. We are hopeful that the improved version now meets your expectations and is more accessible and comprehensible.

Introduction:

2) More information and references are needed on the following three dimensions: geographical granularity (country vs. regions or departments), the way in which excess mortality is estimated (compared with a past level or with estimated past trends, and in the latter case, which model is used), and the metric used (life expectancy, vs. standardized mortality rates or numbers of deaths). If it's true that your method would also apply to other scales, you choose to represent the results in terms of lost life expectancy. This choice is not insignificant. Indeed, it is not common to see studies estimating life expectancy losses taking into account past trends (e.g. Aburto (2022) simply compared 2020 and 2021 to 2019), and this should be emphasized more. On the other hand, even if your model is sound, well described and tested in a previous study, other models have also been applied in the literature, many of which also estimate uncertainty around predicted mortality, and you should mention these other methods as well.

(a) Geographical granularity: we have addressed this aspect in the revised version of the introduction by conducting a comprehensive literature review. This review informs the reader about previous studies conducted at both the national and subnational levels. Specifically, for the French case, we explicitly specify that our analysis focuses on *départements*, i.e., NUTS 3 level. Additionally, we have included more detailed information and a reference to enhance the reader's understanding of this specific geographical scale.

"To illustrate this approach, we compute sex-specific excess mortality in 2020 for the 95 départements (departments) in metropolitan France, which correspond to the third level of the Nomenclature of Territorial Units for Statistics (NUTS 3) used by Eurostat (Eurostat, 2021). In order to make comparisons and emphasize the significance of the various sources of uncertainty, we have conducted additional analysis at the NUTS 2 level (régions). This allows us to further explore and highlight the differences in uncertainty across different geographical granularity. " (p. 2, l. 64)

(b) Estimation of excess mortality: In essence, excess mortality can be defined as the difference between a baseline, which signifies the mortality level in the absence of a pandemic, and the observed mortality level during the actual occurrence of the pandemic. In this context, two crucial decisions must be made by all researchers: 1) the method for estimating the baseline, and 2) the metric to be employed for calculating the difference in mortality. In our study, about the former aspect, instead of using pre-pandemic mortality as the baseline, we opt to forecast the temporal pre-pandemic trends into 2020 to estimate the mortality level in the absence of COVID-19. We believe this approach is justified by the fact that mortality patterns commonly change over time, and assuming a static pre-pandemic level as the counterfactual scenario might be overly restrictive. Additionally, at the subnational level, pre-pandemic mortality levels could exhibit significant volatility.

Throughout the paper, we have addressed and discussed these considerations

"More thoughtful accounting of the temporal change in mortality via forecasting techniques have been also proposed for estimating excess mortality at regional level." (p. 2, l. 79)

"the mortality level from the 5 pre-pandemic years is used as the theoretical baseline level without the pandemic. Ignoring decreasing trends in mortality, this approach biases the excess mortality estimate downward" (p. 5, l. 194)

"[. . .] mortality levels that would have been observed had pandemic not occurred need to be estimated. Thus, forecasting methods are necessary to extrapolate temporal mortality variations." (p. 7, l. 283)

And we have also added an additional paragraph in the Introduction to emphasize the importance of these aspects.

"Concerning the first issue, estimating baseline mortality in the absence of COVID-19 by extrapolating pre-pandemic trends is crucial for two primary reasons. In general, assuming a static pre-pandemic level as the counterfactual scenario can be overly restrictive. Moreover, at the subnational level, pre-pandemic mortality may exhibit considerable volatility, making the subjective decision of selecting the number of pre-pandemic years as the baseline particularly challenging." (p. 2, l. 41)

(c) Metric used: We acknowledge the importance of the chosen metric in representing the results and its implications for interpretation. As you rightly pointed out, life expectancy losses are just one of several options that researchers can consider when measuring the impact of the pandemic. Our manuscript does not intend to prioritize one metric over others. Instead, our focus is on highlighting the significance of forecasting mortality during the pandemic using pre-pandemic trends and employing a robust methodology. Additionally, we emphasize the importance of quantifying uncertainty from various sources, irrespective of the metric chosen to describe the burden of the pandemic. In other words, our primary objective is to present a comprehensive and rigorous approach to analyzing the pandemic's impact on mortality while accounting for uncertainties.

Throughout the paper, we have highlighted the versatility and generality of our approach.

"For simplicity, we illustrate the procedure for calculating excess mortality measured by life expectancy at birth, e_0, but the entire process can be customized and applied to other demographic indicators." (p. 4, l. 123)

"we provide derivations to obtain excess mortality estimates and their associated uncertainty when measured by life expectancy at any given age x, age-standardized death rates, and death toll." (p. 5, l. 165)

However, based on your remarks, we acknowledge that there might have been some lack of clarity in conveying our objectives. Therefore, we added the following paragraph in the Introduction to ensure that these aspects are clarified and better communicated.

"Our primary aim is thus to tackle all these challenges within a clear-cut and unified framework. To achieve this, we present a novel approach for estimating subnational excess mortality during pandemics. In essence, our approach is designed to handle all the mentioned issues and provide estimates and uncertainty quantification of the pandemic's burden, regardless of the chosen metric for measuring excess mortality and the geographical granularity of the available data." (p. 2, l. 48)

Regarding the selected forecasting model, we have addressed this in detail in our response to Reviewer #1 (Comment 2). In particular, a new section has been added to the Supporting Information that compares the utilized CP-splines with the conventional Lee-Carter model in terms of excess mortality and classic demographic aspects, such as trends in life expectancy and age-patterns.

Furthermore, we have provided additional explanations and elements to better comprehend CP-splines within the manuscript. These additions can be found in our response to Comment 5 from Reviewer #2.

3) Please refrain from ditirambic statements about the model you've chosen, such as the one in line 52: "we present a fully analytic procedure with enormous advantages from computational cost and time perspective". What is a "fully analytic procedure"?

We acknowledge that our statement may have appeared overly emphatic. As a result, we have decided to rephrase it for better clarity and balance.

"we present an efficient analytic procedure that offers significant advantages in terms of computational cost and time." (p. 2, l. 58)

Regarding your second remark, we have taken your feedback into account and removed the adverb "fully" from our statement. However, we want to emphasize that our approach does not involve any simulation or bootstrap procedure. Instead, we utilize matrix algebra to represent the factors included in the excess mortality measures, and the delta method is employed to estimate their variances. This advantageous feature allows us to avoid computationally consuming procedures, which we have highlighted in another sentence in the paper.

"Instead of following time-consuming simulation and bootstrap procedures, we develop an analytic construction of the variance associated with both observed and forecasted mortality indicators by using delta method." (p. 4, l. 141)

Methods:

4) The prediction model used is a Poisson model including splines for age and period, with some constraints on the coefficients of the spline basis vectors (CO-splines) to avoid aberrant prediction behavior. Given that the method comes from a previous study (Camarda, 2019), it is definitely not useful to go into so many details in the method Section. For example, on line 81, "let arrange the complete matrix in column vectors ....". I would also avoid formulas as the one in equations (2-4), which are at the time too complex and necessarily incomplete, as they rely for example on a (mysterious) matrix L : "constructed in order to select 2020 forecast mortality from (1)". Since all these technical details have previously been described in previous papers, I strongly recommend to simply refer to the literature for more detailed explanations.

We must admit that we had some reservations in fully incorporating this particular remark, as it seems to deviate from the requests made by the other reviewers. Furthermore, we believe that this paper primarily serves as a methodological piece, and including a few clear-cut equations could greatly enhance the reader's understanding of the subsequent steps.

Specifically, equations (2) and (4) play a crucial role in defining how excess mortality is measured in matrix algebra terms and enable us to analytically compute its variance. On the one hand we need to present available data as they are, in matrix form over age and

time. On the other, the model expressed in vectorized forms permits us to incorporate everything within a classic regression framework. This facilitates the derivation of measures such as life expectancy directly from the overall age-time fitted mortality surface.

Anyway, we acknowledge that the formulation of matrix $\boldsymbol{L}$ might have appeared somewhat obscure. In response to this concern, we have taken action to explicitly define it in the Supporting Information (see revised Section A) providing more clarity on the matter. Additionally, we have made edits to the paper to highlight this change.

"Its explicit form is presented in the Supporting Information (Section A)." (p. 4, l. 131)

Lastly, it is worth noting that in the original paper where CP-splines were introduced (Camarda, 2019), a bootstrap procedure was utilized to compute confidence intervals. In contrast, we have made a methodological advancement by employing the delta method with the explicit variance-covariance matrix from the fitted model. This enhancement allows for a more efficient computation of uncertainty around excess mortality measures, and we believe it is essential to acknowledge this progress.

5) Again, avoid some ditirambic statements about the model, as in line 77: "This method allows us to simultaneously estimate and forecast mortality within a regression setting with enormous advantages in the computation of uncertainty measures". I would remind that every prediction method will give simultaneously estimation and predictions of future mortality, and any Poisson model will allow to estimate uncertainty around predictions, which is certainly not a special feature of CP-splines.

We recognize that our choice of words may have seemed excessive or overly complimentary. In response to this feedback, we have taken your suggestion into consideration and have made appropriate revisions to the formulation in the revised paper.

"This method enables the simultaneous estimation and forecast of mortality within a regression setting. Its main advantage lies in a single variance-covariance matrix that encompass uncertainty on both past and future mortality." (p. 3, l. 93)

Nevertheless, we would like to highlight here that alternative mortality forecasting methods generally involve at least two steps. For instance, taking the Lee-Carter model and its variants as an example, future mortality is obtained by first modeling the observed age-time mortality surface to extract a time-index. Then, in a separate step, a time series analysis is applied to the estimated time index. Extrapolated values of this time index, along with the previous parameter vectors, is finally used to produce future mortality age-patterns. The two-step procedure has implications for variance estimation, as uncertainty about future mortality is often derived solely from the variability of the time index, without taking into account the model uncertainty from the first step. See a detailed description of these aspects in Section 4.8 of Basellini et al. (2023).

Lastly, we recognize that CP-splines are not the only model within a Poisson regression setting and we would like to clarify that our paper does not explicitly claim that. However, CP-splines are distinct from most other mortality forecasting methods that utilize the Poisson distribution for the stochastic part. Unlike these methods, CP-splines can be explicitly expressed as a Poisson (penalized) Generalized Linear Model. In the paper, we rather emphasize this last aspect and that due to this feature we can harness all the advantages of this well-established family of models.

"The estimation process follows a classic regression Poisson setting, but with an additional discrete penalty to ensure smoothness of the coefficient vector $\boldsymbol{\alpha}$ *and, consequently, smoothness of mortality* $\boldsymbol{\mu}$*"* (p. 3, l. 102)

"Unlike other methods, using CP-splines offers the advantage of providing an analytical expression for the variance–covariance matrix, $\text{cov}(\boldsymbol{\alpha})$*."* (p. 4, l. 145)

6) I think it's a good idea to use cross-validation to select the time window for estimation. However, why set the minimum window at 2010-2018/19? I'd also like to see what range is obtained for this window across the departments.

Thanks for this comment. In our case, we believe that a minimal time horizon is required to produce accurate forecasts, which, otherwise, would not be unduly affected by random annual variations caused by the small size of the *départements*. In this response letter, we have included a map depicting the starting year of the time-window used to forecast male mortality in 2020 for each *département*. However, after careful consideration, we have decided not to include this map in the paper as it does not reveal any clear spatial pattern. Instead, we have added a single sentence in the main text to provide a broad idea of these results.

"Due to reduced gains in life expectancy since the beginning of the 2000s, the time-window used to forecast male mortality in 2020 starts between 2005 and 2010 for 56 out of 95 départements*, and between 2000 and 2004 for 12 others."* (p. 6, l. 215)

Data and applications

7) First, I would call this section "Results".

We have changed the title accordingly.

8) Figure 1 is quite interesting as an example. However, I would limit myself to the two lower panels, which give your complete results for this department. Here, the comparison with a factual pre-pandemic level is actually interesting, but I'd prefer to see it on the lower panels, where the 95%CI around life expectancy loss actually includes the loss relative to the 2015-19 period. This should be highlighted. I'd also prefer a comparison with 2019,

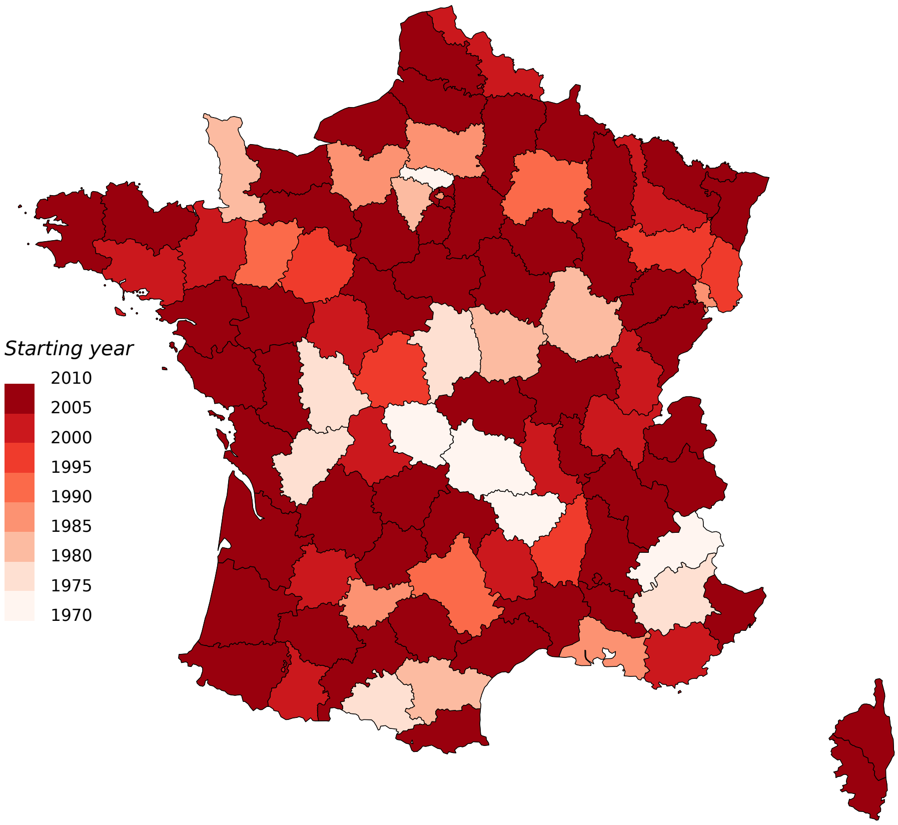

Figure 1: Optimized starting year of the time-window for each *département* utilized, up to 2019, to forecast male mortality in 2020.

rather than 2015-19, since the former choice is the most widely used in the literature (and the most intuitive, given the trend of increasing life expectancy). Then, such a comparison with previous levels is not, in my view, a "naive" comparison, but rather a "factual" one and, in that sense, an (equally) respectable choice. For example, one can state that life expectancy in France in 2020 has fallen by around half a year compared to 2019 (this is not naive, it's just factual), and add that, compared to the trend in life expectancy before the pandemic, the loss has been around 9 months.

Thank you for your comment. After conducting an extensive literature review, we came to the realization that the level of uncertainty surrounding the estimates was frequently overlooked. However, considering this uncertainty is particularly crucial when analyzing results at a very fine spatial level, such as the French *départements*. For pedagogical purposes, we have chosen to retain the four panels in Figure 1 to highlight the two sources of uncertainty and their differentiated impact on our estimates.

Furthermore, it is true that the comparison with 2019 is a common approach used to estimate the excess mortality linked to COVID-19 in 2020, particularly in national-level studies. In our specific case, we have observed substantial annual fluctuations, which can be mainly attributed to the small number of deaths at this geographical level (labelled Poisson uncertainty). As a consequence, relying solely on the year 2019 for comparison may result in highly biased and volatile estimates. To address this issue, we have chosen

to adopt a five-year period (2015-2019) in our comparison. This decision helps to mitigate fluctuations arising from small numbers and aligns our approach with the current literature on the subject.

While we retain our comparison with the 2015-2019 period, we acknowledge the relevance of your comment regarding the label "naive." In response to this valuable feedback, we have made the decision to revise the text, plots, and tables, replacing "naive" with the term "simple".

9) Figures 2 and 3 are, for me, the ones that give interest to the paper. Separating the two sources of variability (the model and the data variability) in Figure 2 is a nice way of representing the results. I'd like to see more description of these figures in the text. I'd also like to see the same figures for women and a comparison between the two sexes and between regions in the results section. Can one see (approximately) the same order of the departments for the two sexes (as it seems reasonable) ? Again, in Figure 2 please replace the comparison to 2015-19 with one to 2019, much more effective.

Many thanks for your kind comment. In line with your demand, we have tried to provide a more in-depth analysis of the results presented in Figures 2 and 3 (p. 6, l. 238). Moreover, we have decided to replicate Figures 2 and 3 for females in the Supporting Information, and added sentences to better describe the differences of the results between the two sexes (p. 6, l. 250).

10) Figure 4 doesn't add much to the results. The messages of these figure can easily be summarized in the text.

As clarified in Comment 8, the literature frequently overlooks the level of uncertainty surrounding the estimates, although accounting for this uncertainty becomes particularly vital when examining results at a very fine spatial level.

We firmly believe that this figure plays a pivotal role in helping readers grasp this concept effectively. Moreover, it facilitates the estimation of the population level where uncertainty is less than x years, thanks to the ample number of *départements* and *régions* in our panel. Describing this relationship succinctly in words can be challenging, and the figure serves as a visual and effective aid in conveying this concept.

11) Figure 5 is interesting for me, since it shows how a larger spatial granularity can hidden more fine variability. However, it should be placed and commented in the Results section.

This paper introduces a novel methodology for estimating excess mortality caused by a pandemic in subnational areas, with French *départements* as illustrative example. The

analysis includes separate estimates for males and females. Additionally, for enhanced understanding, we emphasize the uncertainty surrounding these results.

In the Discussion section, we explore the possibility of reducing this uncertainty by reaggregating populations based on gender or geographical level. Figure 5, which illustrates the case of geographic reaggregation, is particularly pertinent in this context, providing valuable insights for further analysis and understanding.

12) I understand that you're estimating several models, one for each department and gender. However, at least for France and perhaps for some "extreme" departments such as Ile de France and Bretagne, I'd really like to see in one (or more) additional figure(s) the fit of the model at the scale of life expectancy (at age 60), with the observed series, the estimated series and their projections for 2020. Especially when using quite complex and adaptive methods, the fit of a model remains one important piece of information.

We acknowledge that plotting observed, estimated, and forecasted trends can be valuable in assessing the model's performance. In response to Comment 2 from Reviewer #2, we have decided to conduct a comparison between the proposed CP-splines and the more conventional Lee-Carter model. As part of this comparison, we have included plots showcasing life expectancy at age 60 and age-patterns in 2020. Instead of selecting larger spatial units where we obtained smaller differences between the two models in terms of excess mortality, we have chosen to present outcomes for the four *départements* where the differences in excess mortality were estimated to be the largest.

For a comprehensive description of this comparison and the associated figures, please refer to section B of the new Supporting Information. We believe that this additional analysis can fill in the missing piece of information about the model's performance, which you (and possibly a PLOS One reader) may have been looking for in the previous version of the paper.

13) In addition, it would be extremely important to report in the Annexes tables, not only the estimated life expectancy losses in each department, but instead: 1) the life expectancy in 2019, 2) the life expectancy in 2020, 3) the predicted life expectancy in 2020, 4) 2020-2019 (instead of what you call "naïve" comparison w-r-t 2015-2019) and 5) 2020 - predicted 2020 (with your confidence intervals). Was the life expectancy level quite uniform across departments in 2019? Is the loss depending on the initial level? Are results similar for men and women?

We have added life expectancy at age 60 observed in 2015-2019, life expectancy at age 60 observed in 2020 as well as life expectancy forecast in 2020 in our Supporting Information for males, females and both sexes combined (Tables C1, C2, C3)

Discussion

14) A more in-depth discussion of your results for the French departments should be added, with a comparison between regions and sexes, and some attempts to explain the observed variability.

Many thanks for your comment. We have added few sentences to discuss in a more extensive way our results. Please refer to our response to Comment 3 from Reviewer #1

15) Since all your results are based on life expectancy (losses) at age 60, a few words on life expectancy at birth should be added. Is there any change in your results when considering life expectancy at birth?

The pandemic had little impact on the population under 60; in France, mortality rates even fell for the population below age 25 in 2020 (Allard et al., 2020). We have therefore focused our analysis on older people, and chosen to analyze life expectancy at age 60. Yet, our calculations are replicable for life expectancy at birth, and we have ensured that the results are consistent at departmental level.

References

Allard, T., Bayardin, V., and Mosny, E. (2020). L'île-de-france, région la plus touchée par le surcroît de mortalité pendant le confinement. *Insee Analyses*, 118.

Basellini, U., Camarda, C. G., and Booth, H. (2023). Thirty years on: A review of the Lee-Carter method for forecasting mortality. *International Journal of Forecasting.*, 39:1033–1049.

Camarda, C. G. (2019). Smooth Constrained Mortality Forecasting. *Demographic Research*, 41(38):1091–1130.

Currie, I. D., Durbán, M., and Eilers, P. H. C. (2004). Smoothing and Forecasting Mortality Rates. *Statistical Modelling*, 4:279–298.

Eilers, P. H. C. and Marx, B. D. (1996). Flexible Smoothing with B-splines and Penalties (with discussion). *Statistical Science*, 11:89–102.

Eurostat (2021). Nomenclature of territorial units for statistics (nuts). https://ec.europa.eu/eurostat/web/nuts/background. Accessed: 2023-07-27.

Le Minez, S. and Roux, V. (2021). 2020: une hausse des décès inédite depuis 70 ans. *Insee Première*, page 1847.

Lee, R. D. and Carter, L. R. (1992). Modeling and Forecasting U.S. Mortality. *Journal of the American Statistical Association*, 87:659–671.

Preston, S. H., Heuveline, P., and Guillot, M. (2001). *Demography. Measuring and Modeling Population Processes.* Blackwell.
